# Frequency-Guided Cross-Modal Feature Fusion for Lightweight Visible–Infrared Small Object Detection

**DOI:** 10.3390/s26206490

**Published:** 2026-10-13

**Authors:** Zonghuan Chen, Yuheng Li

**Affiliations:** 1 School of Information Engineering, Chang’an University, Xi’an 710018, China; 2025900916@chd.edu.cn; 2 Key Laboratory of Internet Information Retrieval of Hainan Province, Hainan University, Haikou 570228, China

**Keywords:** visible–infrared detection, small object detection, cross-modal fusion, frequency-aware attention, lightweight neural networks, multispectral perception

## Abstract

Visible–infrared detection improves perception in degraded illumination by combining visible texture with thermal contrast. Small objects remain difficult because their discriminative responses are weak, spatially sparse, and sensitive to cross-modal displacement. We present FGCFF-Net, a lightweight frequency-guided fusion detector for visible–infrared small object detection. The framework casts fusion as progressive evidence selection. A lightweight channel pruning module suppresses redundant dual-stream responses; frequency-guided alignment estimates residual spectral compensation; a cross-modal alignment and small-target framework predicts bidirectional spectral weights and deformable offsets; and a frequency-aware cross-modal aggregator conditions channel-spatial attention on frequency discrepancy. The training objective further couples dense detection loss with spectral consistency, offset smoothness, and small-target emphasis. On FLIR, M3FD, LLVIP, and KAIST, FGCFF-Netachieved 92.4% mAP@0.50 on FLIR, 90.7% mAP@0.50 on M3FD, 98.5% AP@0.50 on LLVIP, and 5.72% log-average miss rate on KAIST, with 14.8M parameters and 68 FPS. Together, the results show that frequency-aware alignment can improve multispectral small-object detection without requiring a large model.

## 1. Introduction

Robust object detection in unconstrained road scenes depends on sensors that remain informative when target appearance changes. Visible cameras capture color, lane texture, traffic markings, and fine contours, but their responses degrade under glare, rain, low light, backlight, and saturation. Infrared cameras provide complementary thermal contrast and often reveal pedestrians, vehicles, and riders when color cues collapse. They may also lose semantic texture, suffer from blooming around hot sources, and blur boundaries between thermally similar objects. Effective visible–infrared perception therefore requires local reliability estimation rather than a simple increase in sensor count.

This requirement is most stringent for small objects. A distant pedestrian, rider, bicycle, or traffic-light-related object may occupy only a few cells in a feature map. Even a minor registration error can move the discriminative response away from the detector’s effective sampling region. Many fusion networks address this problem by duplicating branches or applying high-capacity attention at several pyramid levels. These strategies improve representation, but they can amplify redundant background responses and increase deployment cost. Small visible–infrared targets therefore create a coupled requirement: the detector must preserve weak high-frequency details, correct cross-modal misalignment, and remain compact enough for real-time inference.

Existing multispectral detectors have addressed parts of this problem from different directions. Illumination-aware and cross-modal attention methods learn when visible or infrared features should dominate [1,2,3,4]. Transformer-based fusion methods further improve long-range interaction and modality calibration [5,6,7,8]. Recent lightweight and illumination-guided approaches have begun to reduce computation while preserving multispectral robustness [9,10,11,12,13]. Nevertheless, most pipelines still regard alignment, fusion, and detection as loosely connected operations. Cross-modal registration is often handled in the spatial domain alone, while small-target cues are treated by generic feature pyramids or anchor settings. The frequency structure of a target, which directly reflects edge sharpness, thermal spread, and small-object saliency, remains underused.

**Figure 1:**
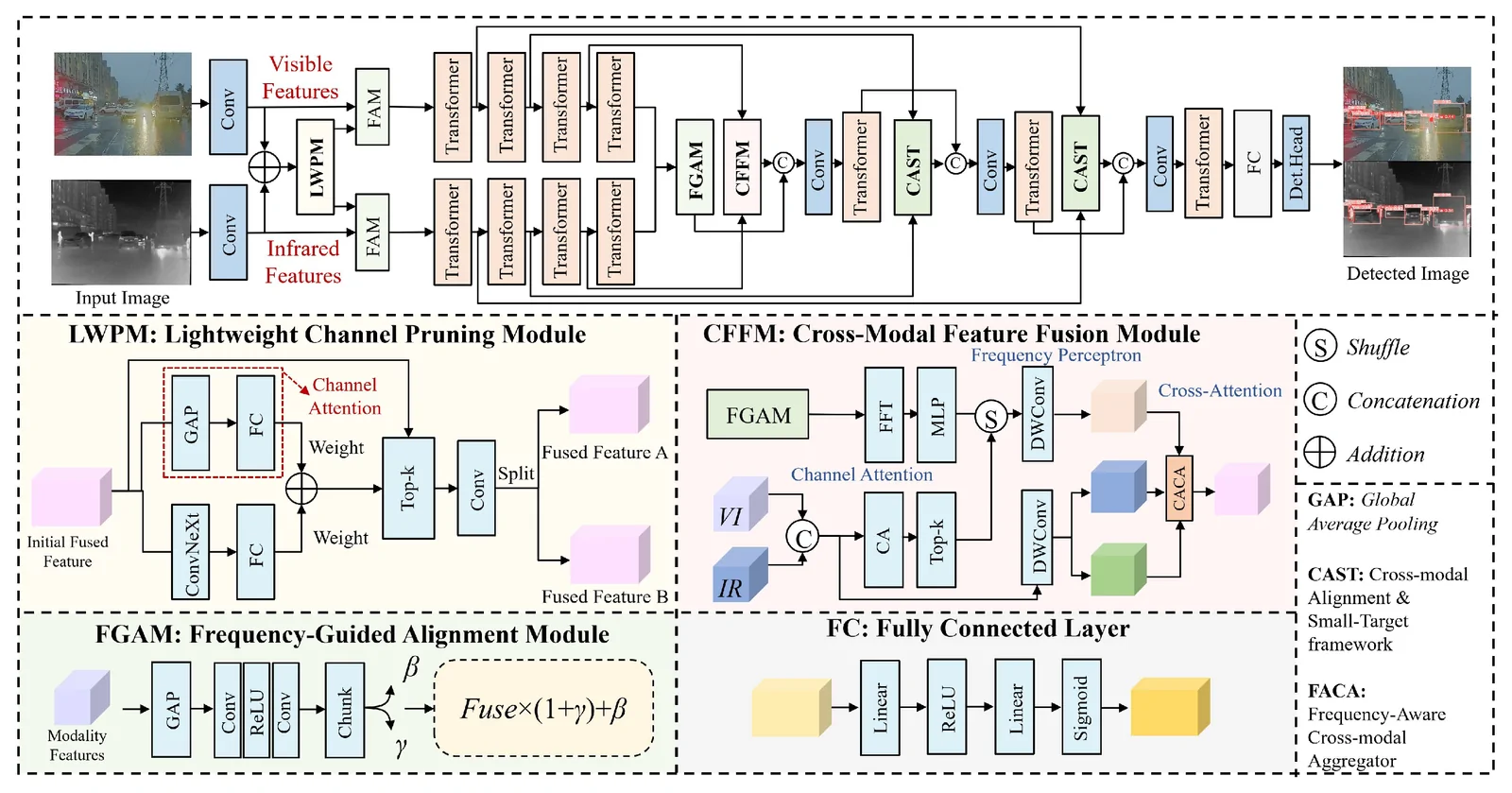
Overview of FGCFF-Net. The framework first extracts visible and infrared features, performs lightweight channel pruning, aligns the modalities through frequency-guided modulation, aggregates calibrated evidence using cross-modal attention, and predicts detections from the fused representation. LWPM removes redundant channels, FGAM provides residual spectral compensation, CAST combines spectral weights and deformable offsets for small-target alignment, and FACA performs frequency-aware cross-modal aggregation. Arrows indicate feature flow; rectangular blocks denote processing modules and cuboids denote feature tensors. Dashed borders group related processing stages. Circled C, S, and plus symbols denote concatenation, channel shuffling, and element-wise addition, respectively. Block colors distinguish operations or feature tensors and do not encode numerical values.

We propose FGCFF-Net, a lightweight detector in which frequency guidance organizes cross-modal fusion. The design is based on the premise that small targets are easier to detect when redundant channels are suppressed before cross-modal interaction, spectral discrepancy is used for compensation, and calibrated responses are aggregated with frequency-aware attention. Figure 1 summarizes this information path. Early dual-stream features are pruned into compact feature responses. Bidirectional frequency modulation estimates how visible and infrared responses should compensate each other. Deformable alignment then samples spatially consistent small-target regions, and the final aggregator merges frequency, channel, and spatial cues before prediction.

The main contributions are as follows. First, we introduce a frequency-guided visible–infrared detection framework that integrates lightweight pruning, spectral alignment, deformable offset prediction, and cross-modal aggregation for small-object detection. Second, we design a Frequency-Guided Alignment Module (FGAM) that learns residual scale and bias terms from spectral descriptors, enabling bounded modality compensation. Third, we develop a Cross-modal Alignment and Small-Target framework (CAST) and a Frequency-Aware Cross-modal Aggregator (FACA) to model bidirectional spectral weights, deformable sampling offsets, channel attention, and spatial attention. Fourth, we formulate a small-target-aware training objective and evaluate the detector on FLIR [14], M3FD [15], LLVIP [16], and KAIST [17].

The remainder of the paper is organized as follows. Section 2 reviews multispectral detection, small-object detection, and frequency-aware representation. Section 3 defines the task and presents LWPM, FGAM, CAST, FACA, and the training objective. Section 4 describes the datasets, evaluation subsets, and implementation protocol. Section 5 reports the main comparisons, controlled ablations, error analysis, scale-wise results, and sensitivity tests. Section 6 and Section 7 discuss the physical and practical limits of the method and summarize the conclusions.

## 2. Related Work

### 2.1. Visible–Infrared Detection

Early multispectral detection work established that visible and thermal images should be processed jointly rather than as independent detections. The KAIST benchmark and its baseline detector provided a widely used evaluation setting for aligned color-thermal pedestrian detection [17]. Subsequent convolutional methods explored early, middle, and late fusion, often using two-stream backbones and shared detection heads. Illumination-aware Faster R-CNN adapted modality weighting to lighting conditions [1], CIAN introduced cross-modality interactive attention [2], and AR-CNN addressed weak spatial alignment between sensors [3]. MBNet further argued that visible and infrared streams can become imbalanced during training and proposed balancing strategies to reduce modality dominance [4].

More recent work has shifted from static fusion toward adaptive and transformer-based interaction. Cyclic fuse-and-refine and guided attentive fusion blocks improved repeated exchange between modalities [18,19]. CFT introduced transformer-style cross-modality fusion [5], ICAFusion used iterative cross-attention to refine multispectral features [6], C^2^Former calibrated complementary cues with a transformer design [7], and CAGTDet added cascade alignment guidance [8]. Parallel efforts, including MOD-YOLO, M^2^FNet, GMD-YOLO, MFMGNet, MMI-Det, mask-guided multi-level fusion, VIF-YOLO, intermediate fusion, and PMoE, emphasized lightweight detector backbones, adaptive modality routing, or more explicit multi-level interaction [9,10,20,21,22,23,24,25,26]. These studies show that cross-modal attention is useful, but they also reveal a remaining gap: high-capacity attention can improve global fusion while still missing small targets if misalignment and high-frequency degradation are not explicitly controlled.

Recent studies have also widened the design space beyond task-specific two-stream detectors. UniRGB-IR adapts a frozen visible-light foundation model to several RGB–IR semantic tasks through lightweight feature-pooling and injection modules [27]. E2E-MFD jointly optimizes image fusion and object detection in a single training process [28], while adaptive cross-modal fusion explicitly adjusts visible and infrared contributions under changing imaging conditions [29]. Multiview infrared representation learning provides a complementary perspective by learning view-consistent object features without paired visible imagery [30]. These methods motivate broader forms of modality and view adaptation. Our study focuses more narrowly on compact feature alignment and aggregation for paired visible–infrared small-object detection.

### 2.2. Small Object Detection and Efficient Detectors

Small object detection is difficult because the object signal is easily overwhelmed by stride, pooling, background clutter, and label assignment noise. Surveys have grouped the main remedies into multiscale representation, context modeling, super-resolution, and proposal design [31,32,33]. Modern detectors have strengthened these components through feature pyramids [34], dense classification losses [35], end-to-end transformers [36,37,38], and real-time one-stage designs such as SSD, YOLO variants, Gold-YOLO, YOLOv9, YOLOv10, and RT-DETR [39,40,41,42,43,44,45,46,47]. These detectors provide strong baselines for single-modality or generic object detection, but visible–infrared small objects introduce an additional coupling: scale and modality reliability change together. A distant pedestrian at night may be clear in infrared but weak in visible, while a reflective vehicle edge may be clearer in visible but thermally ambiguous. Efficient multispectral detection therefore requires a mechanism that is both scale-sensitive and modality-aware.

### 2.3. Frequency and Attention in Multimodal Representation

Frequency channel attention has shown that channel reweighting can encode spectral preferences with modest computational overhead [48]. Deformable convolution provides another important ingredient by allowing the sampling grid to adapt to object geometry and spatial displacement [49]. In visible–infrared detection, recent methods have begun to exploit edges, illumination priors, frequency mining, semantic guidance, bidirectional masking, deformable prompting, and hierarchical feature flow [11,12,50,51,52,53,54,55]. Broader infrared-visible fusion reviews also indicate that neural fusion quality depends on preserving both low-frequency structure and high-frequency detail [56]. The present work builds on these observations but targets detection rather than image reconstruction: frequency descriptors are not used to make a visually pleasing fused image; they are used to decide how object evidence should be aligned, weighted, and aggregated for detection.

## 3. Materials and Methods

### 3.1. Terminology and Physical Scope

Table 1 defines the terms used throughout the manuscript. Here, cross-modal fusion refers to feature-level exchange between paired visible and infrared streams, rather than pixel-level image synthesis. Frequency guidance denotes the use of feature-spectrum descriptors to condition alignment or attention. It does not imply that the detector reconstructs a fused image.

The two sensors are affected by different image-formation processes. We use the following compact model to state the physical scope of the method:

(1) $$I^{m}=D_{m}\;\left(h_{m}*L^{m}\right)+n_{m},\;m\in \left\{v,i\right\},$$

where $h_{m}$ is the modality-specific point-spread function, $D_{m}$ denotes sampling and sensor response, and $n_{m}$ collects readout, shot, and quantization noise. In the visible band, $h_{v}$ includes defocus and monochromatic aberrations that blur edges or displace local energy. Atmospheric transmission is approximated by the Bouguer–Lambert relation

(2) $$\tau_{m}\left(\lambda ,d\right)=\exp\left[-\kappa_{m}\left(\lambda \right)d\right],$$

where $\kappa_{m}$ is the wavelength-dependent extinction coefficient and *d* is the propagation distance. For the thermal datasets used here, the received infrared radiance also depends on target emissivity, temperature-dependent emission, reflected environmental radiance, and detector noise. Consequently, glare can saturate visible responses, whereas hot backgrounds, thermal blooming, and sensor noise can reduce infrared contrast. Occlusion affects both streams geometrically and cannot be removed by spectral compensation alone. The proposed network learns feature-level reliability under these effects, but it does not estimate optical parameters or solve radiative transfer explicitly.

### 3.2. Problem Formulation

Given a registered or weakly registered visible image $I^{v}\in R^{H\times W\times 3}$ and an infrared image $I^{i}\in R^{H\times W\times 1}$, the detector predicts a set of bounding boxes $B={\left\{\left(b_{n},c_{n},s_{n}\right)\right\}}_{n=1}^{N}$, where $b_{n}$ is the bounding box, $c_{n}$ is the category label, and $s_{n}$ is the confidence score. A lightweight dual-stream stem extracts modality features, followed by a shared pyramid ${\left\{P_{l}\right\}}_{l=3}^{5}$. The index starts at l=3 because $P_{3}$, $P_{4}$, and $P_{5}$ are the detector pyramid levels with strides 8, 16, and 32. It is not a count of the proposed modules, and $P_{1}$ and $P_{2}$ are omitted from the head to control memory and preserve the standard detection hierarchy. Unless a dataset supplies an official size label, we follow the resized-image rule in Table 1: small objects have area below $32^{2}$ pixels and tiny objects have area below $16^{2}$ pixels. Object intensity is evaluated separately as a contrast or imaging-condition factor and is not part of the geometric size definition.

For each pyramid level, the processing chain is written as a composition of explicit stage functions:

(3) $$\begin{array}{cc} Z_{l}^{\left(0\right)} & =\Phi_{\mathrm{LWPM}}\left(F_{l}^{v},F_{l}^{i}\right),\\ Z_{l}^{\left(1\right)} & =\Phi_{\mathrm{FGAM}}\left(Z_{l}^{\left(0\right)}\right),\\ Z_{l}^{\left(2\right)} & =\Phi_{\mathrm{CAST}}\left(Z_{l}^{\left(1\right)}\right),\\ P_{l} & =\Phi_{\mathrm{CFFM}}\left(Z_{l}^{\left(2\right)}\right),\;B=H\left({\left\{P_{l}\right\}}_{l=3}^{5}\right). \end{array}$$

Here, $\Phi_{\mathrm{LWPM}}$ denotes lightweight channel pruning, $\Phi_{\mathrm{FGAM}}$ denotes frequency-guided alignment, $\Phi_{\mathrm{CAST}}$ denotes cross-modal alignment and small-target sampling, $\Phi_{\mathrm{CFFM}}$ denotes the cross-modal feature fusion module built around FACA, and H is the detection head. The modules are ordered so that expensive cross-modal interaction is applied only after early channels have been filtered and spectrally compensated.

### 3.3. Lightweight Channel Pruning Module

Dual-stream networks often double feature channels before the model has determined which responses are useful. This is inefficient and can dilute small-target signals. The Lightweight Channel Pruning Module (LWPM) in Figure 1 addresses this issue by generating two complementary channel scores from global and local evidence. For an initially fused feature $F_{0}=\mathrm{Cat}\left(F^{v},F^{i}\right)$, LWPM computes

(4) $$\omega_{g}=\sigma \left(W_{2}\delta \left(W_{1}\mathrm{GAP}\left(F_{0}\right)\right)\right),\;\omega_{l}=\sigma \left(W_{4}\delta \left(W_{3}{\mathrm{Conv}}_{3\times 3}\left(F_{0}\right)\right)\right),$$

where δ is ReLU, GAP extracts global channel statistics, and the local branch preserves neighborhood-sensitive evidence. The channel importance score is

(5) $$\omega =\alpha \omega_{g}+\left(1-\alpha \right)\mathrm{GAP}\left(\omega_{l}\right),\;k=\left\lceil rC\right\rceil ,\;S_{k}=\underset{c\in \{1,\ldots ,C\}}{arg topk}\omega_{c},$$

where r∈(0,1] is the keep ratio and *C* is the channel count. Let $P_{S_{k}}$ gather the selected channels. The pruned feature is

(6) $$\begin{array}{cc} F_{p} & ={\mathrm{Conv}}_{1\times 1}\;\left(P_{S_{k}}\left(F_{0}⊙\omega \right)\right),\\ \left(F_{p}^{v},F_{p}^{i}\right) & =\mathrm{Split}(F_{p}). \end{array}$$

The hard TopK index operation is not differentiable. During a forward pass, its indices are treated as fixed. Gradients flow through the gathered feature values and the selected multiplicative scores in Equation (6), but not through changes in the discrete index set. No straight-through estimator is used. LWPM is therefore piecewise differentiable between ranking changes, rather than fully differentiable with respect to TopK. Ties are resolved by the deterministic channel order of the implementation. The split operation partitions the projected feature along the channel dimension into two compact tensors used by the subsequent modality branches.

### 3.4. Frequency-Guided Alignment Module

Small visible–infrared targets are often recognizable through local high-frequency patterns such as pedestrian contours, vehicle edges, and thermal boundaries. However, the relative strength of these patterns changes across modalities. FGAM therefore models alignment in the frequency domain and returns bounded residual modulation coefficients for feature compensation. For a modality feature $F_{l}^{m}$, m∈{v,i}, its frequency representation is

(7) $${\hat{F}}_{l}^{m}=F\left(F_{l}^{m}\right)=A_{l}^{m}\exp\left(j\Theta_{l}^{m}\right),$$

where $A_{l}^{m}$ and $\Theta_{l}^{m}$ denote amplitude and phase. We construct a compact spectral descriptor by pooling low-, middle-, and high-frequency bands:

(8) $$\begin{array}{cc} z_{l}=\mathrm{Cat}( & \mathrm{GAP}\left(M_{\mathrm{low}}⊙A_{l}^{v}\right),\mathrm{GAP}\left(M_{\mathrm{mid}}⊙A_{l}^{v}\right),\mathrm{GAP}\left(M_{\mathrm{high}}⊙A_{l}^{v}\right),\\ \; & \mathrm{GAP}\left(M_{\mathrm{low}}⊙A_{l}^{i}\right),\mathrm{GAP}\left(M_{\mathrm{mid}}⊙A_{l}^{i}\right),\mathrm{GAP}\left(M_{\mathrm{high}}⊙A_{l}^{i}\right),\\ \; & \mathrm{GAP}(|A_{l}^{v}-A_{l}^{i}|)). \end{array}$$

where $M_{\mathrm{low}}$, $M_{\mathrm{mid}}$, and $M_{\mathrm{high}}$ are disjoint masks that partition the feature spectrum into low-, middle-, and high-frequency regions. The descriptor is mapped to modality-specific residual scale and bias terms:

(9) $$\left[\gamma_{l}^{m},\beta_{l}^{m}\right]=\mathrm{Chunk}\left(\psi_{\mathrm{fgam}}^{m}\left(z_{l}\right)\right),\;{\tilde{F}}_{l}^{m}=F_{l}^{m}⊙\left(1+\tanh\left(\gamma_{l}^{m}\right)\right)+\beta_{l}^{m},\;m\in \left\{v,i\right\}.$$

The tanh constraint prevents the scale term from overwhelming the original feature, which is important when one modality is temporarily unreliable. FGAM thus behaves as an alignment-aware residual adapter rather than a hard modality selector.

### 3.5. Cross-Modal Alignment and Small-Target Framework

**Figure 2:**
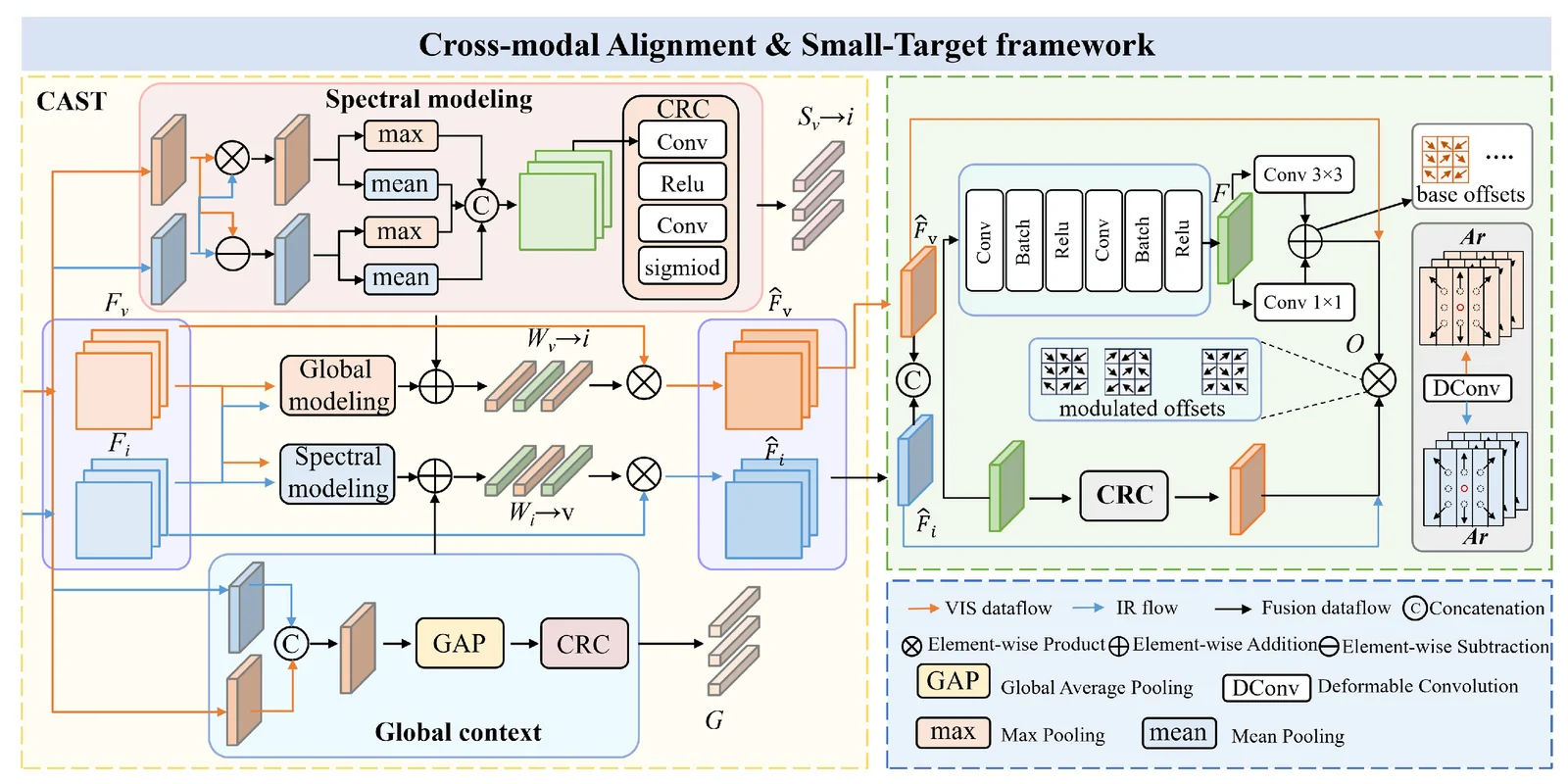
Cross-modal Alignment and Small-Target framework (CAST). Spectral modeling estimates bidirectional modulation weights, global context stabilizes modality compensation, and deformable convolution uses modulated offsets to align small-target evidence in the spatial domain. Orange and blue streams denote visible and infrared feature flow, respectively; black arrows denote fused-feature flow. Stacked planes depict feature tensors. Circled C, multiplication, plus, and minus symbols denote concatenation, element-wise multiplication, addition, and subtraction, respectively. Dashed arrows connect the predicted modulation to the sampling offsets. CRC denotes the convolution–ReLU–convolution sequence, followed by sigmoid activation where shown; GAP denotes global average pooling and DConv deformable convolution.

CAST, illustrated in Figure 2, converts spectral reliability into spatially adaptive sampling. The left part performs bidirectional spectral modeling. Given modality features $F_{l}^{v}$ and $F_{l}^{i}$, max and mean spectral descriptors are concatenated and passed through a channel recalibration cell (CRC):

(10) $$S_{v\to i}=\psi_{\mathrm{crc}}\left(\mathrm{Cat}(\mathrm{MaxPool}\left({\hat{F}}_{l}^{v}⊙{\hat{F}}_{l}^{i}\right),\mathrm{MeanPool}\left({\hat{F}}_{l}^{v}-{\hat{F}}_{l}^{i}\right))\right),$$

with an analogous $S_{i\to v}$. These descriptors produce directional weights $W_{v\to i}$ and $W_{i\to v}$, which compensate each modality before concatenation:

(11) $${\bar{F}}_{l}^{v}=F_{l}^{v}⊙W_{i\to v}+G_{l},\;{\bar{F}}_{l}^{i}=F_{l}^{i}⊙W_{v\to i}+G_{l},$$

where $G_{l}$ is a global context vector shared by both streams.

The right part of CAST estimates offsets for small-target alignment. From the concatenated compensated feature ${\bar{F}}_{l}=\mathrm{Cat}\left({\bar{F}}_{l}^{v},{\bar{F}}_{l}^{i}\right)$, the offset branch predicts

(12) $$\Delta p_{l}={\mathrm{Conv}}_{3\times 3}\left({\bar{F}}_{l}\right)+{\mathrm{Conv}}_{1\times 1}\left({\bar{F}}_{l}\right)+B_{l},$$

where $B_{l}$ denotes base offsets and the residual convolutions produce modulated offsets. The aligned feature at location *p* is

(13) $$F_{l}^{\mathrm{align}}\left(p\right)=\sum_{q\in R}w_{q}\cdot {\bar{F}}_{l}\left(p+q+\Delta p_{l,q}\right),$$

where R is the convolutional sampling grid and $w_{q}$ is the learned kernel weight. This design links cross-modal reliability to the sampling positions used by the detector, which is especially helpful when a small infrared hotspot and its visible contour are slightly displaced.

### 3.6. Frequency-Aware Cross-Modal Aggregator

**Figure 3:**
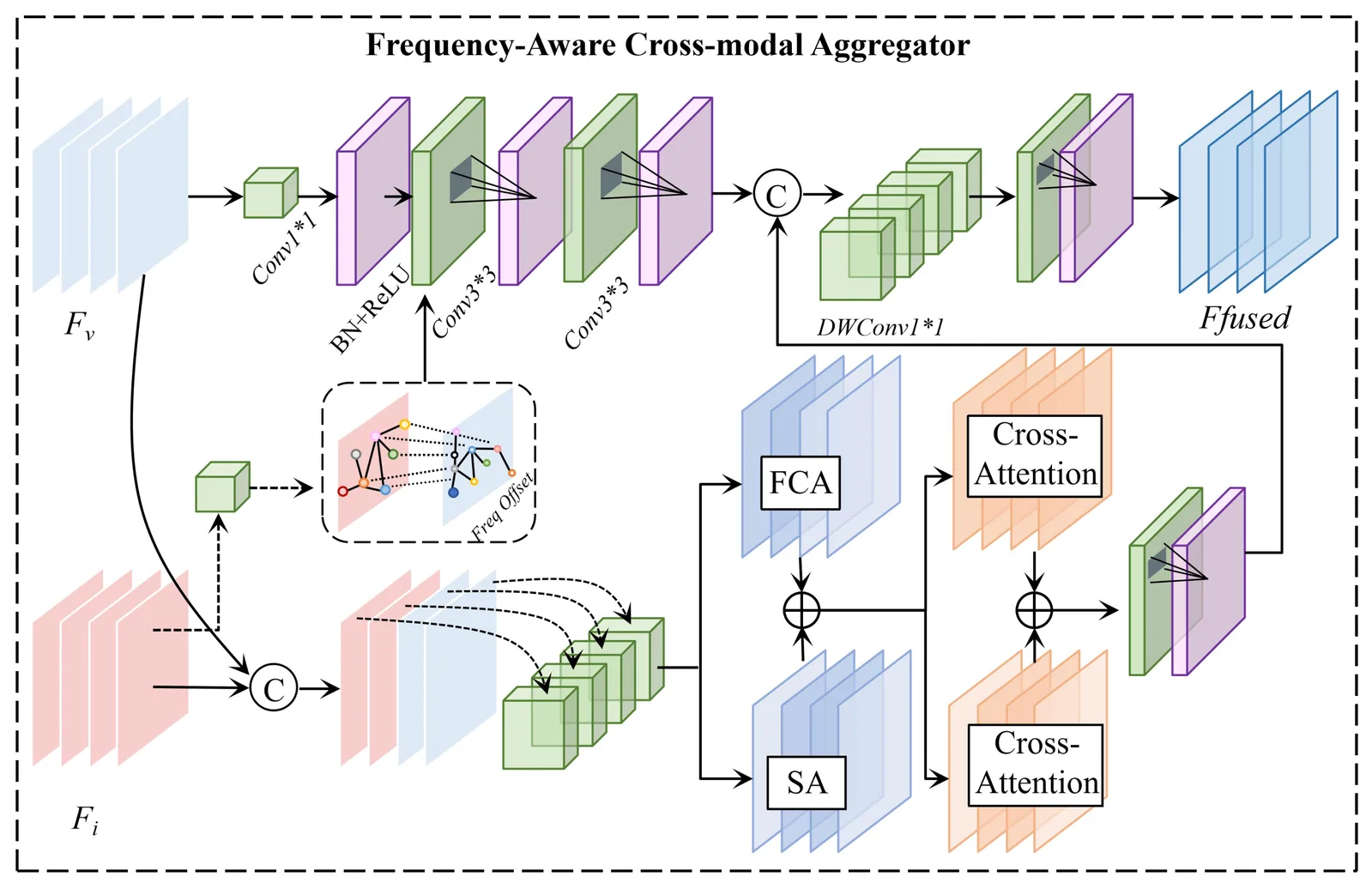
Frequency-Aware Cross-modal Aggregator (FACA). Frequency offsets derived from visible–infrared spectral discrepancy modulate channel and spatial cross-attention before the fused feature is processed by depthwise convolution. Blue and pink input planes represent visible and infrared feature tensors, respectively. Green blocks/planes represent convolutional feature transformations; purple planes denote normalization and activation stages. Blue attention blocks denote frequency channel attention (FCA) and spatial attention (SA), and orange blocks denote cross-attention. Solid arrows show feature flow and dashed arrows the frequency-offset/conditioning path. Circled C and plus symbols denote concatenation and element-wise addition. The asterisk in kernel labels separates the kernel dimensions: 1*1 and 3*3 mean 1×1 and 3×3, respectively. BN denotes batch normalization.

After CAST has aligned the modalities, the model still needs to aggregate features without washing out small-target details. FACA, shown in Figure 3, performs this step by injecting frequency offsets into channel-spatial cross-attention. The visible branch first produces a query-like representation through 1×1 and 3×3 convolutions, while the infrared branch contributes complementary key-value evidence. A frequency offset embedding $E_{f}$ is generated from the spectral discrepancy:

(14) $$E_{f}=\rho \left(\mathrm{GAP}\left(|{\hat{F}}_{l}^{v}-{\hat{F}}_{l}^{i}|\right)\right),$$

and is added to the attention logits:

(15) $${\mathrm{Attn}}_{f}\left(Q,K,V\right)=\mathrm{Softmax}\left(\frac{Q{\left(K+E_{f}\right)}^{⊤}}{\sqrt{d}}+\Pi_{l}\right)V,$$

where $\Pi_{l}$ denotes a positional bias. The channel branch FCA(·) and the spatial branch SA(·) provide complementary views:

(16) $$F_{l}^{\mathrm{fused}}={\mathrm{DWConv}}_{1\times 1}\left(\mathrm{Cat}\left({\mathrm{Attn}}_{f}\left(Q_{c},K_{c},V_{c}\right),{\mathrm{Attn}}_{f}\left(Q_{s},K_{s},V_{s}\right)\right)\right).$$

The Cross-Modal Feature Fusion Module (CFFM) is the stage wrapper that combines the CAST-aligned feature with the FACA output:

(17) $$\Phi_{\mathrm{CFFM}}\left(Z_{l}^{\left(2\right)}\right)={\mathrm{Conv}}_{1\times 1}\left(\mathrm{Cat}\left(Z_{l}^{\left(2\right)},F_{l}^{\mathrm{fused}}\right)\right)=P_{l}.$$

This definition closes the composition in Equation (3). Unlike generic cross-attention, FACA makes its attention preference depend on frequency discrepancy. When a visible edge and an infrared hotspot agree, their joint response is reinforced. When they disagree, the residual path in Equation (17) preserves modality-specific evidence instead of forcing an average.

### 3.7. Detection Head and Training Objective

The detection head follows a lightweight decoupled design. Classification and localization branches share the fused pyramid features but use separate convolutional towers to avoid competition between category confidence and precise small-box regression. For a predicted box *b* and ground-truth box $b^{*}$, the base detection loss is

(18) $$L_{\det}=\lambda_{\mathrm{cls}}L_{\mathrm{vfl}}+\lambda_{\mathrm{box}}L_{\mathrm{CIoU}}+\lambda_{\mathrm{dfl}}L_{\mathrm{DFL}},$$

where $L_{\mathrm{vfl}}$ follows the focal-style dense classification principle [35], $L_{\mathrm{CIoU}}$ penalizes box geometry [57], and $L_{\mathrm{DFL}}$ models box boundary distributions [58].

To make the training objective consistent with the proposed architecture, we add three auxiliary terms. The frequency consistency loss reduces destructive spectral disagreement:

(19) $$L_{\mathrm{freq}}=\frac{1}{3}\sum_{l=3}^{5}{∥\mathrm{Norm}\left(M_{\mathrm{obj}}⊙A_{l}^{v}\right)-\mathrm{Norm}\left(M_{\mathrm{obj}}⊙A_{l}^{i}\right)∥}_{1},$$

where $M_{\mathrm{obj}}$ is a soft mask expanded from ground-truth boxes. The offset smoothness loss regularizes deformable sampling around small targets:

(20) $$L_{\mathrm{off}}=\frac{1}{3}\sum_{l=3}^{5}\left(∥\nabla_{x}\Delta p_{l}{∥}_{1}+{∥\nabla_{y}\Delta p_{l}∥}_{1}\right).$$

Finally, the small-target emphasis term assigns higher weight to boxes with smaller relative area:

(21) $$\eta \left(b^{*}\right)=1+\tau \exp\left(-\frac{\sqrt{a\left(b^{*}\right)/\left(HW\right)}}{\mu }\right),\;L_{\mathrm{small}}=\frac{1}{N}\sum_{n=1}^{N}\eta \left(b_{n}^{*}\right)L_{\det}\left(b_{n},b_{n}^{*}\right).$$

The final objective is

(22) $$L=L_{\det}+\lambda_{f}L_{\mathrm{freq}}+\lambda_{o}L_{\mathrm{off}}+\lambda_{s}L_{\mathrm{small}}+\lambda_{r}{∥\omega ∥}_{1}.$$

For any tensor *X*, the entrywise norm is defined as ${∥X∥}_{1}=\sum_{i}\left|X_{i}\right|$. The operators $\nabla_{x}$ and $\nabla_{y}$ in Equation (20) are first-order forward differences along the two spatial axes. Thus, ${∥\omega ∥}_{1}$ regularizes the channel scores, whereas the two $\ell_{1}$ terms in $L_{\mathrm{off}}$ penalize abrupt spatial changes in the predicted offsets.

Algorithm 1 summarizes the complete training procedure.

**Algorithm 1** Training procedure of FGCFF-Net.
   **Input:** Paired images $\left\{I^{v},I^{i}\right\}$, labels Y, parameters θ, and loss weights $\lambda_{f},\lambda_{o},\lambda_{s},\lambda_{r}$.**Output:** Optimized parameters $\theta^{*}$.
    1**for** each training mini-batch **do**    2   Extract ${\left\{F_{l}^{v},F_{l}^{i}\right\}}_{l=3}^{5}$ with the dual-stream backbone.    3   **for** l∈{3,4,5} **do**    4      Compute $\omega_{l}$ and $S_{k}$ and obtain the split feature pair $Z_{l}^{\left(0\right)}$ with LWPM using Equations (4)–(6).    5      Obtain $Z_{l}^{\left(1\right)}$ by FGAM spectral compensation using Equations (7)–(9).    6      Predict bidirectional weights and offsets with CAST, then sample $Z_{l}^{\left(2\right)}$ by Equation (13).    7      Compute frequency-aware attention with Equation (15) and obtain $P_{l}$ by Equation (17).    8   **end for**    9   Predict boxes, classes, and confidence scores with $H({\left\{P_{l}\right\}}_{l=3}^{5})$.     10   Evaluate the five terms in Equation (22).     11   Update θ by back-propagating Equation (22).     12
**end for**
     13**return** $\theta^{*}$.


## 4. Experimental Setup

### 4.1. Datasets

Table 2 summarizes the dataset protocol and evaluation settings.

**Figure 4:**
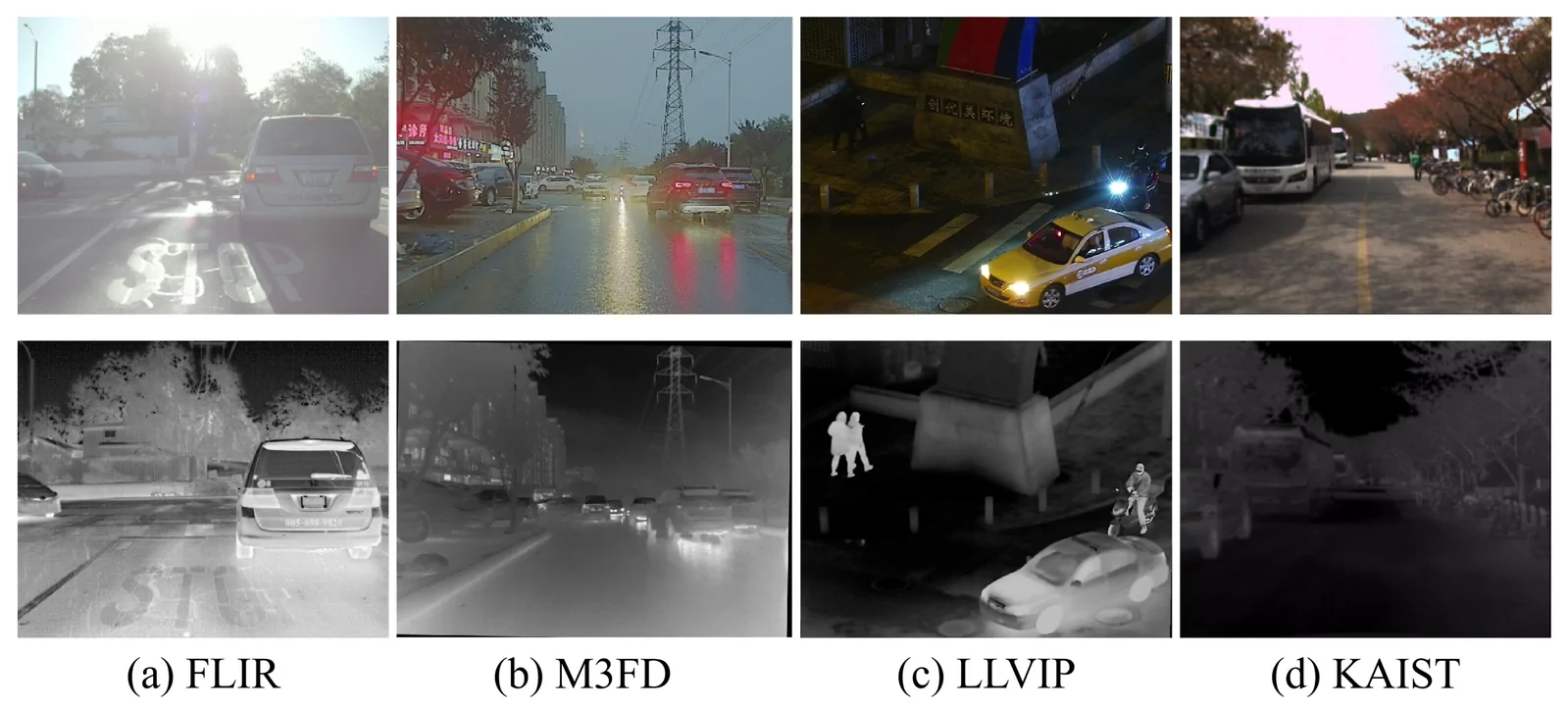
Examples from the four visible–infrared benchmarks used in this study. Each column shows a visible image and its infrared counterpart from FLIR, M3FD, LLVIP, and KAIST.

The experiments use four public visible–infrared datasets that cover different sensing conditions. FLIR contains aligned thermal and visible road scenes with common traffic objects [14]. M3FD provides a multi-scenario benchmark containing visible and infrared pairs for detection-oriented fusion [15]. LLVIP focuses on low-light visible–infrared pedestrian perception [16]. KAIST is a classical multispectral pedestrian dataset collected in urban driving scenes under day and night conditions [17]. Figure 4 shows representative image pairs and indicates why a single fusion rule is insufficient across datasets: FLIR includes glare and small road users, M3FD includes weather and scale variation, LLVIP emphasizes low-light pedestrians, and KAIST contains both urban clutter and cross-modal contrast changes.

### 4.2. Evaluation Subsets and Error Definitions

Condition-specific subsets were formed before model inference and then kept identical for all methods. Day and night followed the official tags where available; otherwise, the label was assigned from ambient illumination in the visible frame. Glare required a saturated or blooming light region that overlapped a target or its immediate context. Rain/fog required visible precipitation streaks or atmospheric veiling in both members of the image pair. Tiny objects followed the geometric threshold in Table 1. Occlusion denoted a target with less than 70% of its estimated full extent visible, based on dataset flags when available and manual review otherwise. Two annotators independently applied the non-geometric labels and resolved disagreements before predictions were inspected.

For the error analysis, a missed small object was an unmatched ground-truth box below $32^{2}$ pixels at IoU <0.5. A duplicate was a second or later prediction of the same class matched to one ground-truth box. A background false positive had a maximum IoU of <0.1 with every ground-truth box. A localization error had the correct class and 0.1≤IoU<0.5. These definitions were fixed before comparing the baseline and the full model.

### 4.3. Implementation Details

All images were resized to 640×640 unless otherwise specified. The detector was trained for 300 epochs using AdamW with an initial learning rate of $1.0\times 10^{-3}$, weight decay of $5.0\times 10^{-4}$, cosine decay, and a warm-up period of 3 epochs. The batch size was 16 on two NVIDIA RTX 4090 GPUs (NVIDIA Corporation, Santa Clara, CA, USA). The implementation used Python 3.11, PyTorch 2.7.1, torchvision 0.22.1, and NVIDIA CUDA 12.6. Data augmentation included mosaic, random horizontal flipping, random scale jitter, color perturbation for the visible branch, and infrared intensity jitter for the thermal branch. For paired images, geometric augmentation was shared across modalities to preserve alignment.

The default loss weights were $\lambda_{\mathrm{cls}}=1.0$, $\lambda_{\mathrm{box}}=7.5$, $\lambda_{\mathrm{dfl}}=1.5$, $\lambda_{f}=0.2$, $\lambda_{o}=0.05$, $\lambda_{s}=0.4$, and $\lambda_{r}=1.0\times 10^{-4}$. The LWPM keep ratio was 0.65, and CAST used a 3×3 deformable sampling grid. These architectural settings and auxiliary-loss weights were fixed across all four datasets; they were selected on the FLIR validation split and were not retuned per test set. Dataset-specific changes were limited to the class count and official split. All module ablations used the same backbone, initialization, augmentation, optimizer, schedule, image size, and evaluation code. Inference speed was measured with batch size one after 200 warm-up iterations and 1000 timed iterations. The one-factor sensitivity tests varied only the stated parameter and retained all other settings.

### 4.4. Compared Methods

The comparison covers single-modality detectors, generic object detectors adapted to two-stream input, convolutional multispectral detectors, transformer-based fusion methods, and recent lightweight or illumination-aware visible–infrared detectors. This design avoids an overly narrow comparison against only one detector family. Table 3 lists the main baselines and their role in the evaluation.

## 5. Results

### 5.1. Main Quantitative Comparison

Table 4 reports the main comparison on FLIR and M3FD.

Across FLIR and M3FD, FGCFF-Netoccupied an accuracy-oriented point in the measured accuracy–efficiency trade-off. The largest margins were observed for AP_S_, which reflects the intended emphasis on weak high-frequency target cues. On FLIR, the detector reached 92.4% mAP_50_ and 47.9% AP_S_, exceeding C^2^Former by 3.0 and 4.1 points, respectively. On M3FD, it achieved 90.7% mAP_50_ with 14.8 M parameters. This performance used fewer parameters than transformer-heavy fusion baselines such as CAGTDet and DMPFDet, although several compact single-modality YOLO baselines retained higher raw throughput.

The comparison also reveals why visible–infrared small-object detection cannot be solved by either a stronger unimodal detector or a heavier fusion block alone. Recent one-stage detectors such as YOLOv9 s and YOLOv10 s offered high inference speed, but their AP_S_ remained limited because thermal saliency and visible texture were not explicitly reconciled. Transformer-based fusion improved the absolute accuracy, yet the improvement was accompanied by larger parameter counts, higher FLOPs, and lower throughput. FGCFF-Netoccupied a different regime: compared with DMPFDet, it improved FLIR mAP_50_ by 1.4 points and M3FD mAP_50_ by 0.9 points, while using 72.7% fewer parameters and running at approximately 2.8 times the speed. The gain is therefore attributable to better cross-modal evidence before aggregation rather than model scale alone.

The LLVIP and KAIST results showed the same overall ranking, although the error sources differed across datasets. LLVIP is dominated by low-light pedestrian detection, where infrared evidence is strong but visible structure remains useful for rejecting background responses. KAIST is more sensitive to day–night variation and weak registration. FGCFF-Netobtained 65.4% AP_S_ on LLVIP and reduced the KAIST log-average miss rate to 5.72%, while also reaching 80.1% mAP_50_ on KAIST.

Table 5 reports the main comparison on LLVIP and KAIST. The magnitude of the improvement differed across the two pedestrian-oriented benchmarks. On LLVIP, most fusion models already achieved high AP_50_ because pedestrians usually appear as clear thermal responses against dark visible backgrounds. The remaining challenge was therefore not only recall but also precise localization at stricter overlap thresholds and small scale. The 0.7-point AP_75_ gain over DMPFDet and the 0.7-point AP_S_ gain indicate that the proposed alignment and frequency aggregation refine the spatial support of pedestrian boxes. On KAIST, the absolute mAP_50_ values were lower, and the miss rate was more discriminative. The reduction from 6.09% to 5.72% MR over DMPFDet, together with a 1.3-point AP_S_ gain, shows that the method remains useful when day–night transitions and imperfect registration make thermal-visible correspondence less stable.

### 5.2. Qualitative Detection Behavior

**Figure 5:**
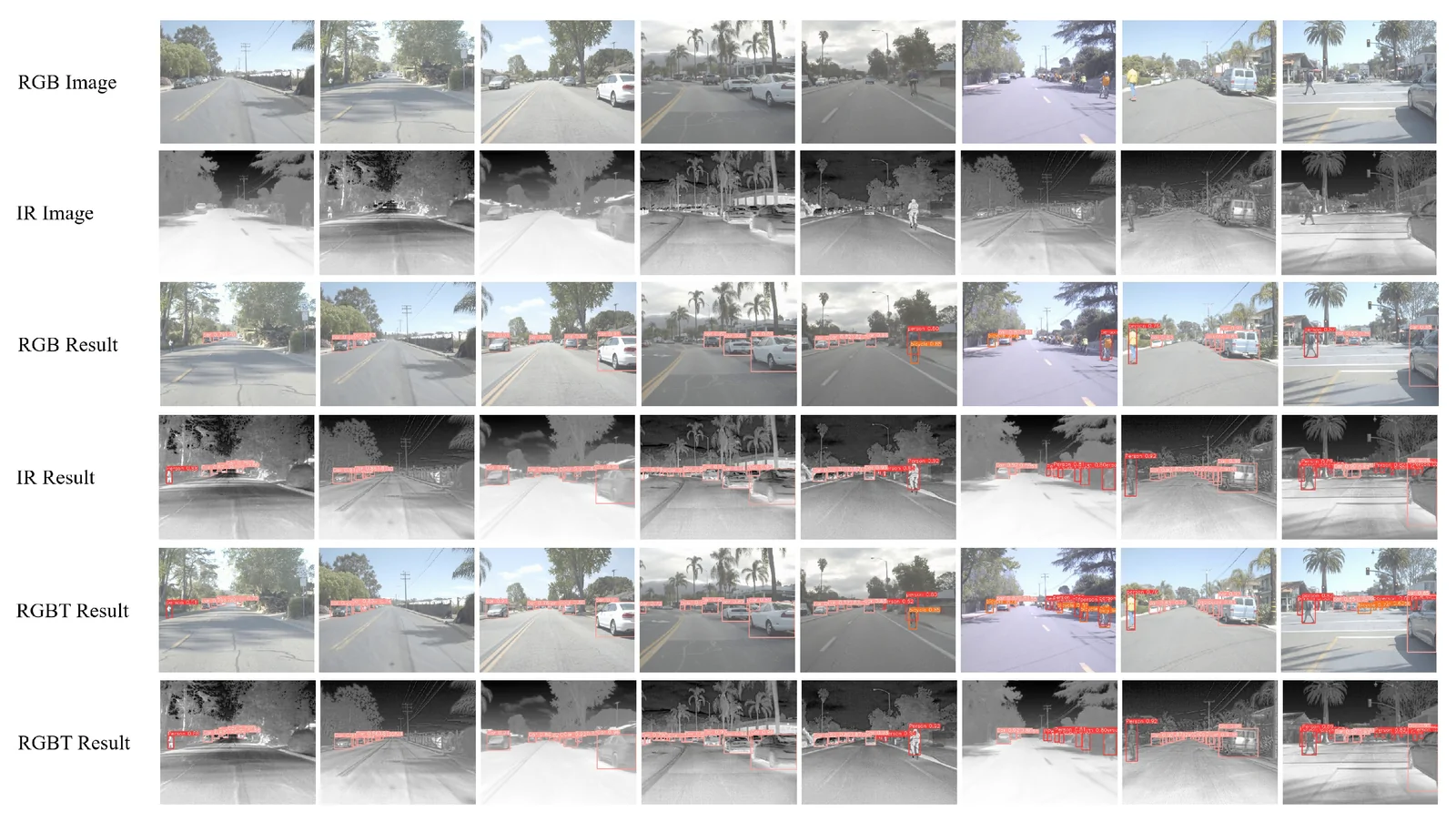
Detection examples on FLIR. Compared with single-modality results, the proposed visible–infrared fusion improves recall for distant pedestrians, vehicles, and riders under illumination variation. Colored rectangles show predicted object bounding boxes; adjacent labels give predicted classes and confidence scores. The box colors are annotation display colors and do not represent confidence or temperature; classes are identified by the text labels.

**Figure 6:**
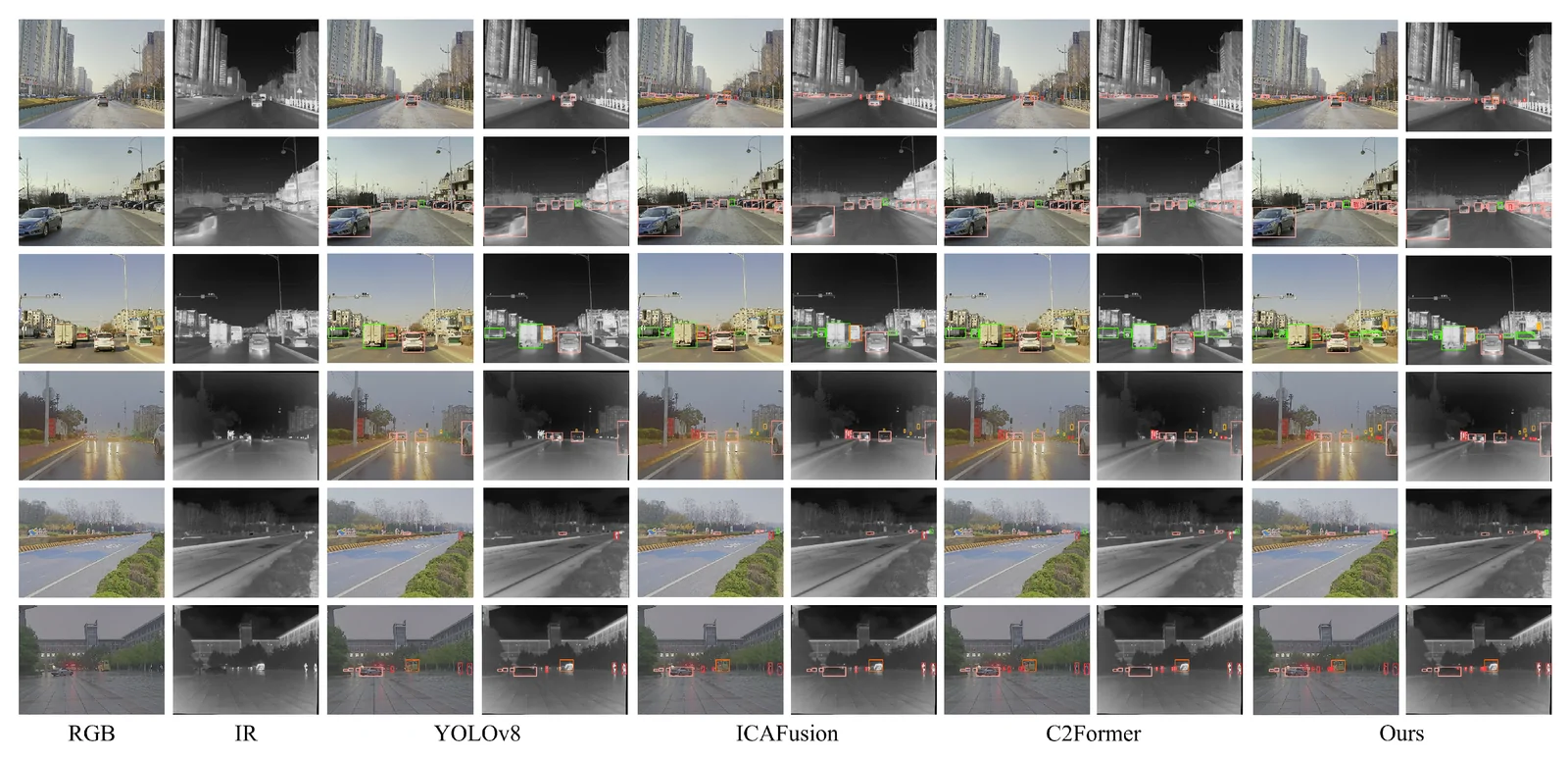
Detection examples on M3FD. The proposed method is compared with YOLOv8, ICAFusion, and C^2^Former under diverse traffic scenes. Rectangles show predicted object bounding boxes; labels, where displayed, give the predicted class and confidence. Box colors are annotation display colors without a quantitative scale.

The qualitative comparisons are consistent with the quantitative results. In FLIR scenes, visible-only predictions missed distant pedestrians in glare or overexposed regions, whereas infrared-only predictions produced false responses around hot background structures. The fused predictions in Figure 5 recovered more small vehicles and pedestrians across both visible and thermal outputs. In M3FD, Figure 6 shows that FGCFF-Netpreserved small road objects in rainy and hazy scenes. These cases include low-contrast targets missed by YOLOv8 and duplicate boxes produced by heavier fusion methods.

Several recurring error patterns were reduced by the proposed design. First, small pedestrians at road boundaries were less likely to disappear when one modality was locally unreliable, because the frequency branch retained contour-level evidence from the complementary modality. Second, vehicle boxes were more stable in scenes with reflections or headlight saturation, where visible features could dominate thermal features if the fusion weights were not calibrated. Third, duplicate detections around adjacent warm structures were reduced, which is consistent with the offset smoothness and spectral consistency terms in the training objective. These observations are important because small-object detection errors are often qualitative before they become visible in aggregate AP: a model may obtain a reasonable mAP while still missing the objects that matter most in safety-critical scenes.

**Figure 7:**
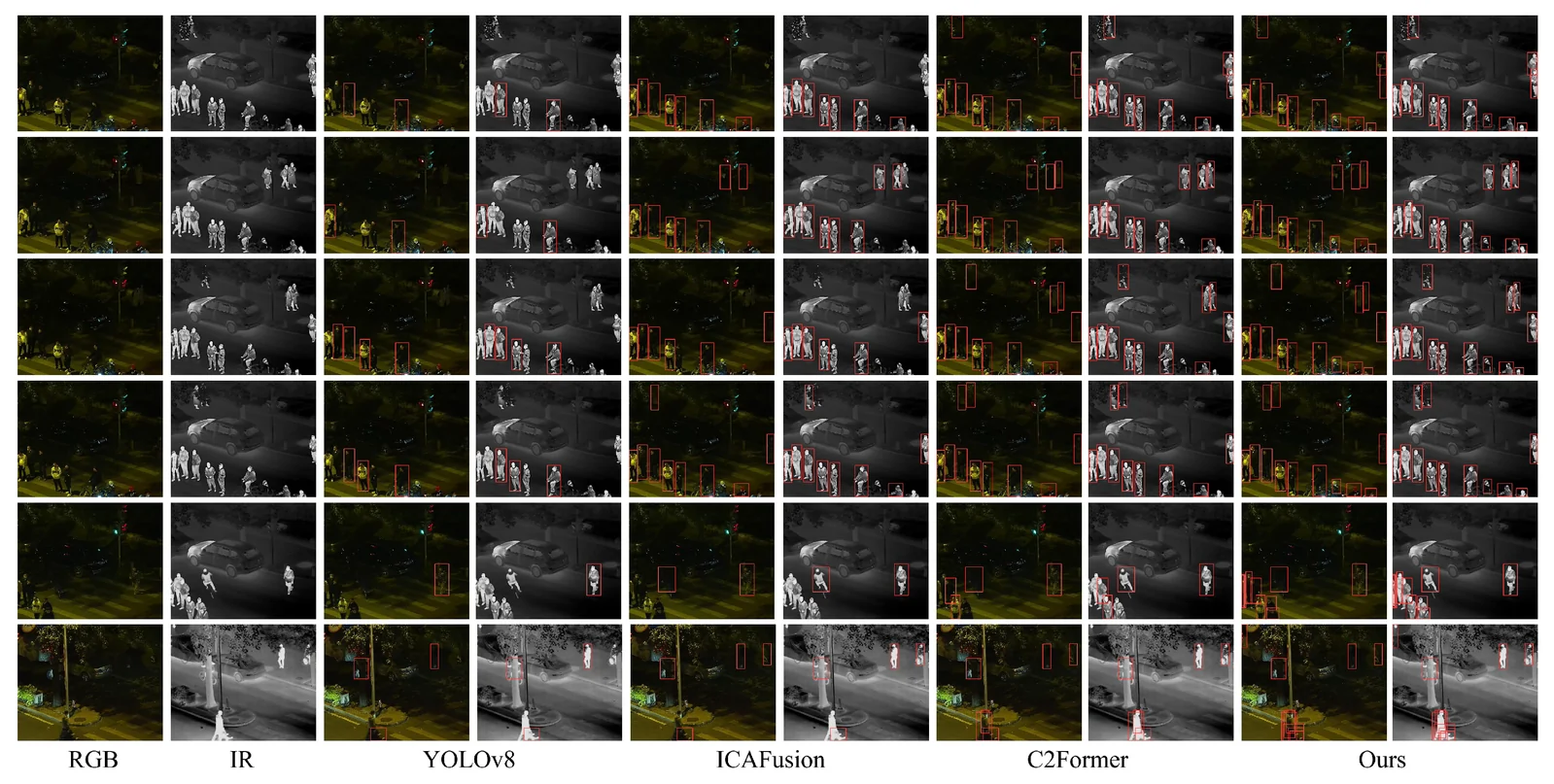
Detection examples on LLVIP. The low-light setting emphasizes the need to fuse thermal saliency with visible structural constraints. Rectangles show predicted object bounding boxes; labels, where displayed, give the predicted class and confidence. Box colors are annotation display colors without a quantitative scale.

**Figure 8:**
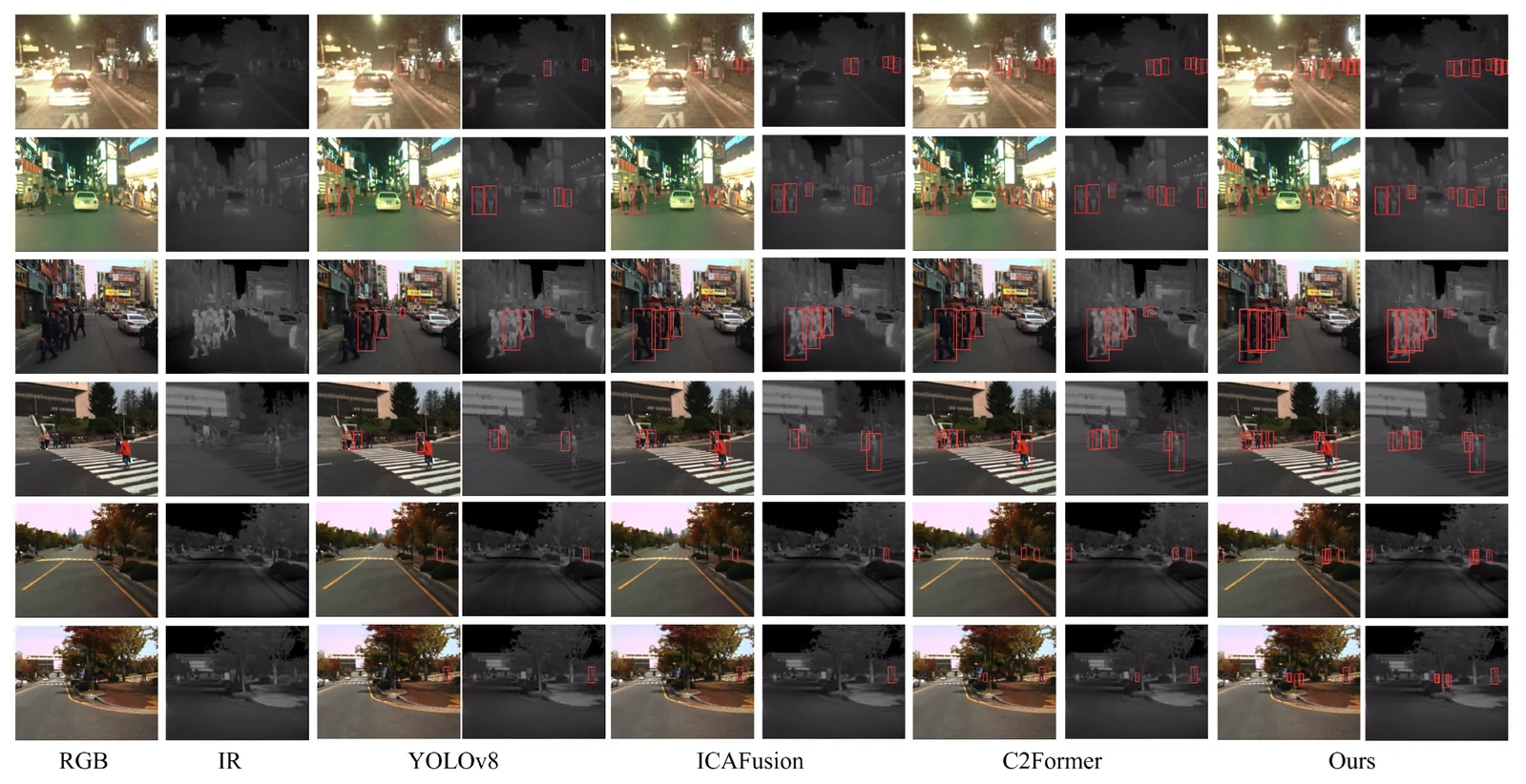
Detection examples on KAIST. The proposed detector improves small pedestrian localization in mixed day and night urban scenes. Rectangles show predicted object bounding boxes; labels, where displayed, give the predicted class and confidence. Box colors are annotation display colors without a quantitative scale.

The LLVIP and KAIST visual results further indicate the role of cross-modal alignment. In LLVIP, many pedestrians appear as strong thermal regions with weak visible texture. As shown in Figure 7, FGCFF-Netmaintained high pedestrian recall while reducing the background activation caused by unconstrained infrared saliency. In KAIST, Figure 8 shows that small pedestrians near bright signs, crosswalks, and road edges were localized more consistently when the deformable offset branch in CAST was used.

The two datasets also illustrate different forms of modality dominance. In LLVIP, infrared features often provide the primary object evidence, but a detector that follows thermal intensity too aggressively may activate on background heat sources. In KAIST, visible features can be informative during daytime, whereas thermal responses are more reliable at night or under shadow. The proposed bidirectional spectral weighting therefore has a practical role: it does not assign a fixed priority to one modality but adjusts the contribution according to local frequency discrepancy and global context. This adaptive weighting allows the same architecture to accommodate datasets with different illumination statistics.

**Figure 9:**
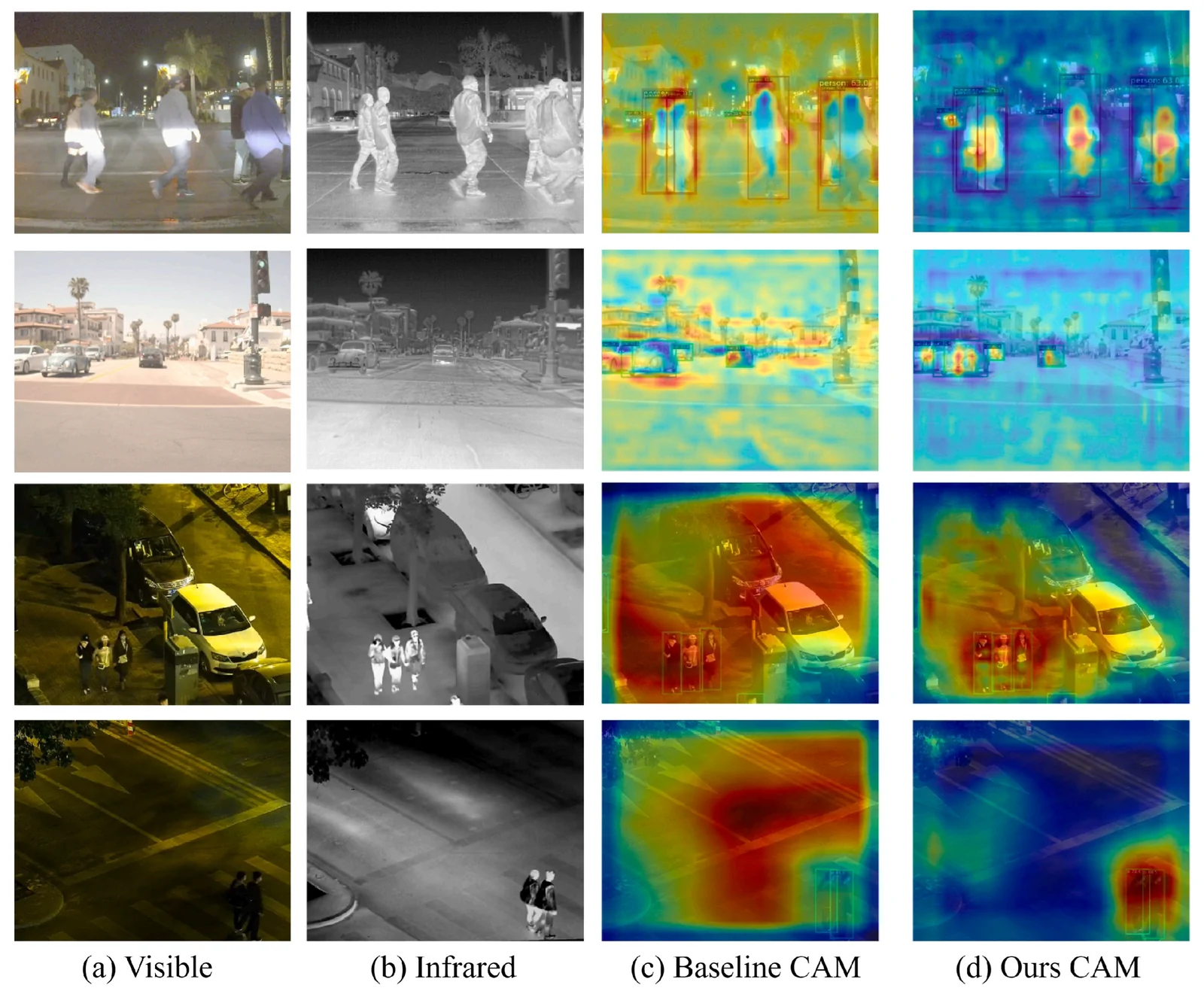
Class activation maps for representative visible–infrared scenes. The proposed model concentrates responses on pedestrians and vehicles more precisely than the baseline. Blue/cyan indicates weaker activation and yellow/red stronger activation within each displayed heatmap; these colors do not represent physical temperature. Rectangular outlines indicate object locations and are separate from the activation color scale.

**Figure 10:**
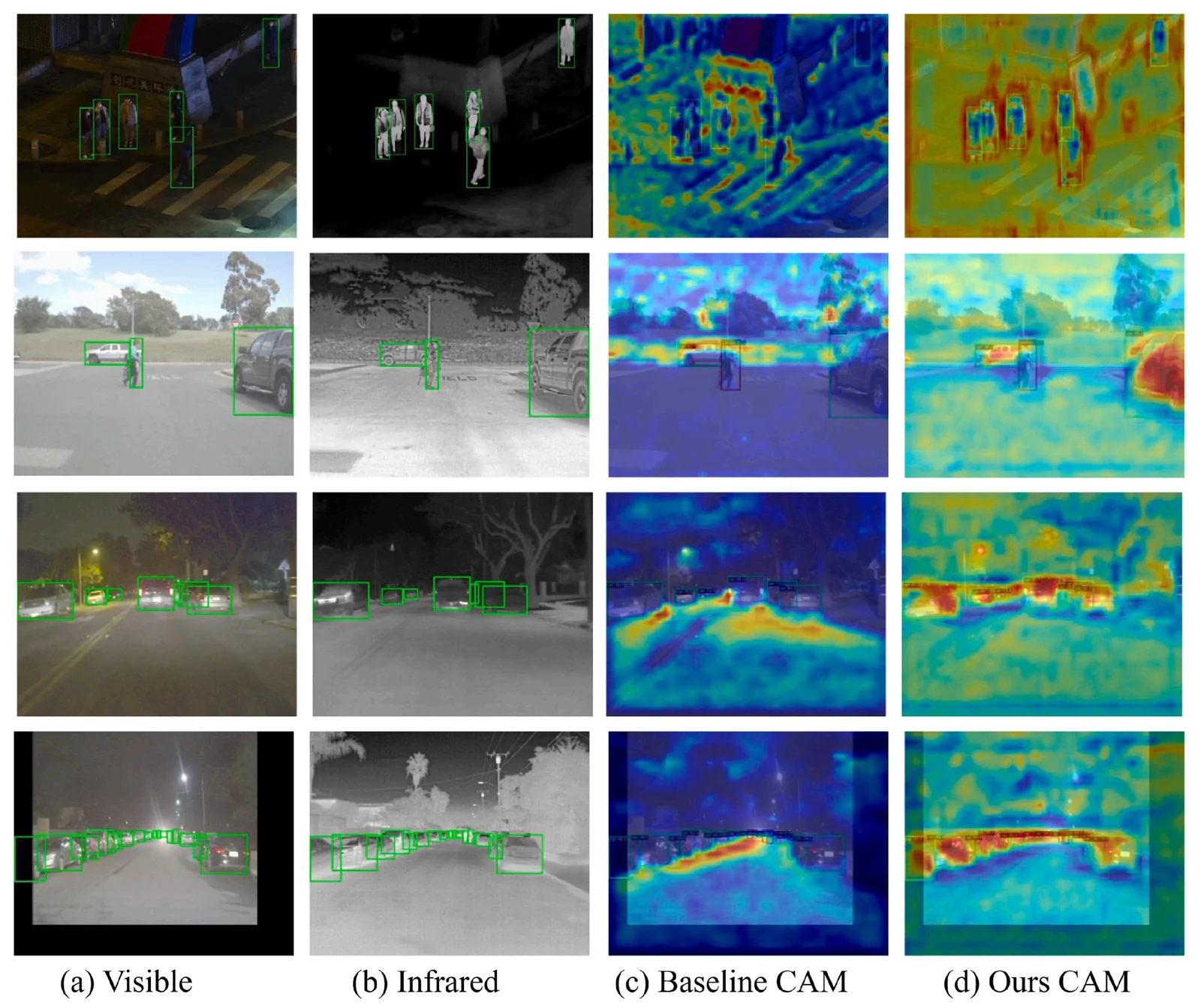
Additional activation maps under small-target, night, and cluttered-road settings. The proposed frequency-aware fusion suppresses broad background activation and improves object-centered attention. Blue/cyan indicates weaker activation and yellow/red stronger activation within each displayed heatmap; these colors do not represent physical temperature. Rectangular outlines indicate object locations and are separate from the activation color scale.

Class activation maps provide a direct view of the fusion behavior. The baseline activation in Figure 9 and Figure 10 spread over road surfaces, lamps, and warm background regions. In contrast, FGCFF-Netconcentrated activation inside object boxes and along target contours. The more compact response agrees with the intended role of frequency guidance: preserving high-frequency target boundaries while reducing modality-inconsistent background energy.

The heatmaps also clarify the division of labor among the proposed components. Frequency modeling sharpened the regions where visible and infrared cues agreed at object boundaries, while deformable alignment reduced the response drift caused by local displacement. The final aggregation stage did not simply increase activation magnitude; it redistributed attention from broad background areas to compact target regions. Stronger activation alone can increase false positives, whereas a compact response is more consistent with improved localization and fewer duplicate detections.

### 5.3. Quantitative Error Analysis

Table 6 complements Figure 5, Figure 6, Figure 7, Figure 8, Figure 9 and Figure 10 with four error categories defined in Section 4.2. Relative to the matched middle-fusion baseline, the full model reduced missed small objects and localization errors on both validation sets. Duplicate and background detections also decreased, which is consistent with the more compact activation patterns in Figure 9 and Figure 10. The table reports normalized counts so that the two datasets can be compared despite their different sizes.

### 5.4. Accuracy–Efficiency Trade-Off

**Figure 11:**
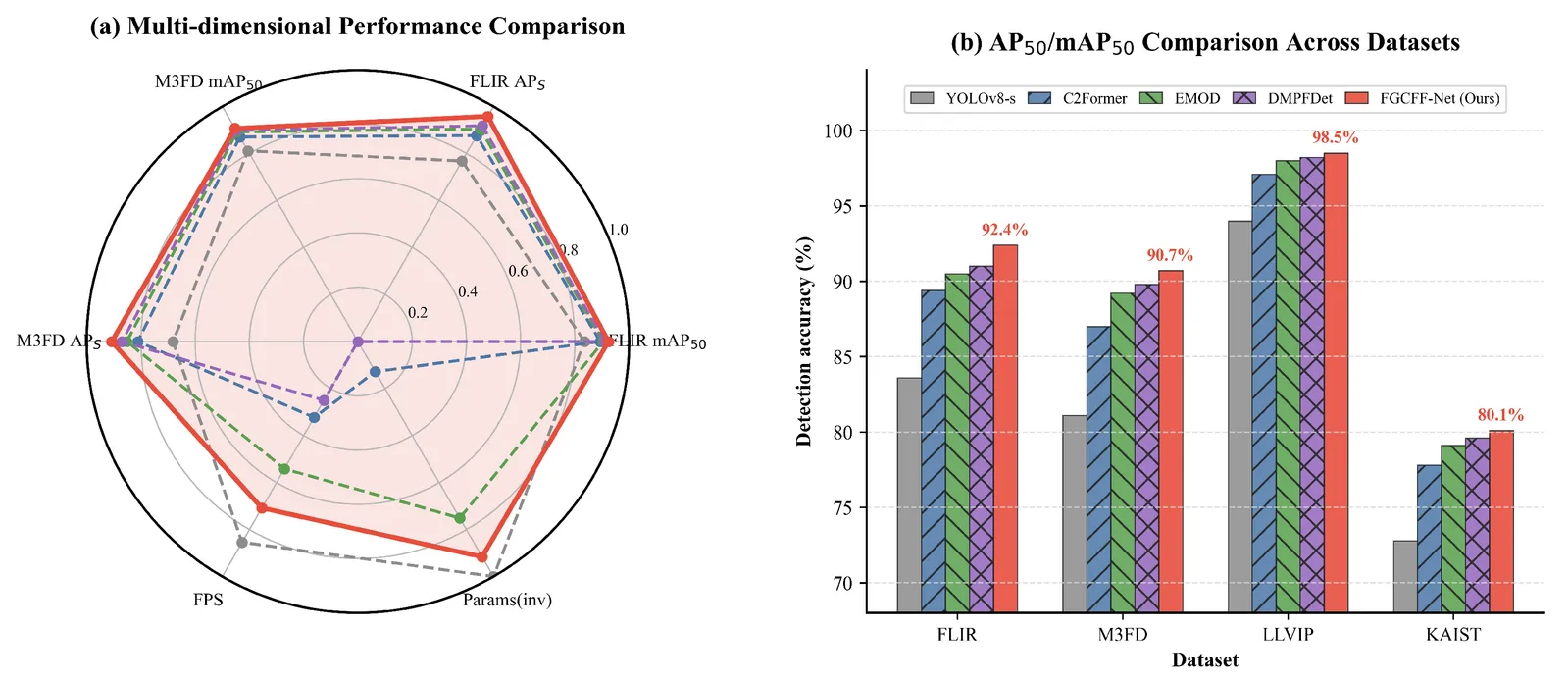
Multi-dimensional comparison among representative visible–infrared detectors. Panel (**a**) reports normalized FLIR/M3FD accuracy, AP_S_, inference speed, and inverse parameter cost. Panel (**b**) compares AP_50_/mAP_50_ on FLIR, M3FD, LLVIP, and KAIST. In (**a**), gray, blue, green, and purple dashed curves denote YOLOv8-s, C^2^Former, EMOD, and DMPFDet, respectively; the red solid curve denotes FGCFF-Net. The same method colors are used for the bars in (**b**).

A practical detector for vehicle or edge sensing should be evaluated by accuracy, small-object sensitivity, computational cost, and cross-dataset stability. Figure 11 therefore summarizes the tabular results from two complementary views. The radar plot places FGCFF-Netin an accuracy-oriented operating point with moderate model size and throughput. It does not have the highest raw FPS. The grouped comparison shows gains on FLIR, M3FD, LLVIP, and KAIST, indicating that the frequency-guided design is not tied to a single illumination distribution.

The computational profile is mainly a consequence of where cross-modal interaction is placed. Early interaction at full channel width is expensive, whereas late interaction may miss small objects after downsampling. LWPM reduces the channel width before FGAM, CAST, and FACA. The resulting 68 FPS is slower than YOLOv5s (96 FPS), YOLOv10 s (88 FPS), and YOLOv8 s (82 FPS), but the proposed model provides higher multispectral accuracy and AP_S_ under the matched protocol. The result should therefore be read as an accuracy–throughput trade-off, not as a claim of maximum inference speed.

### 5.5. Scale-Wise Behavior

The scale-wise comparison in Table 7 separates tiny, small, medium, and large objects using the resized box area. The largest gains occurred in the tiny bin and then narrowed as object size increased on both datasets. Frequency-guided alignment is therefore most useful when a target occupies few pixels. This does not imply that frequency information is unimportant for larger objects; conventional spatial features simply represent them more reliably.

### 5.6. Ablation Study

The ablation results in Table 8 follow the proposed information path under a controlled protocol. Every row used the same backbone, data split, augmentation, optimizer, schedule, image size, random-seed policy, and detection head; only the listed modules or loss terms changed. Starting from a middle-fusion YOLOv8 baseline, LWPM improved accuracy while reducing parameters. Adding FGAM then produced a further gain on FLIR and M3FD. The largest incremental gain in the sequence occurred when FACA was added, and CAST further improved small-object AP and the KAIST miss rate. Because the rows are cumulative, the table estimates incremental contributions within this fixed pipeline rather than all possible module interactions.

The cumulative improvements were monotonic across all four benchmarks. Relative to the middle-fusion baseline, the full model increased FLIR mAP_50_ by 5.6 points, M3FD mAP_50_ by 5.8 points, LLVIP AP_50_ by 3.3 points, and KAIST mAP_50_ by 6.5 points. The average AP_S_ over FLIR, M3FD, and KAIST increased from 37.4% to 44.9%, while KAIST MR decreased from 9.91% to 5.72%. The larger gains on KAIST and on AP_S_ show that the modules are not only increasing overall representation capacity; they are specifically improving the conditions under which small targets and weakly aligned modalities are most vulnerable.

**Figure 12:**
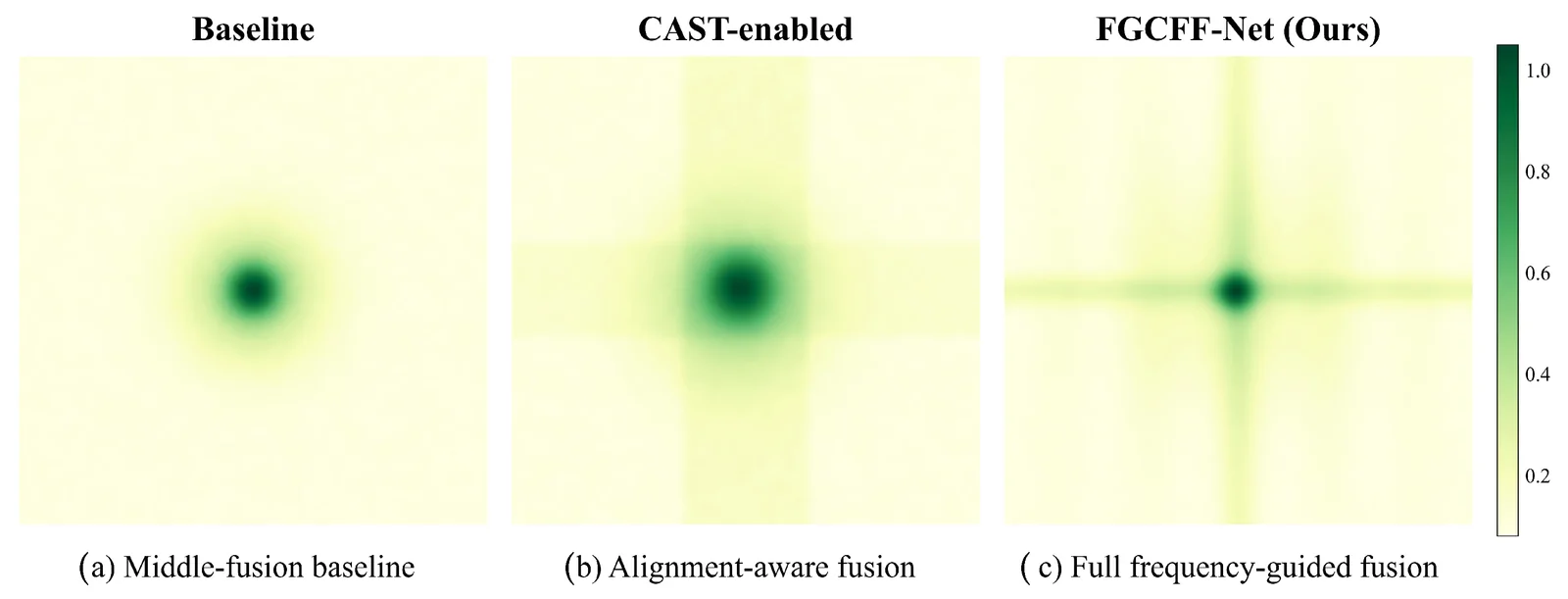
Activation compactness maps for representative ablation states. The full frequency-guided fusion objective produces a sharper object-centered response while retaining structured cross-modal context. Panels (**a**–**c**) show the middle-fusion baseline, CAST-enabled alignment-aware fusion, and the full model, respectively. Pale yellow indicates weaker activation and dark green stronger activation, as indicated by the color bar.

The scalar ablations are also consistent with the response pattern observed in Figure 12. The baseline fusion response is locally activated but remains diffuse around the target center. After alignment-aware fusion is introduced, the response becomes stronger yet still carries residual horizontal and vertical spread. With the full objective, the activation peak is sharper and the surrounding response is more structured, which is consistent with the AP_S_ gains and the lower KAIST miss rate.

### 5.7. Loss Function and Hyperparameter Analysis

Table 9 reports the loss-function ablation. The auxiliary losses improved detection rather than only regularizing intermediate features. Frequency consistency increased AP_S_ more than overall mAP, and adding offset smoothness produced a further gain on both datasets. The small-target emphasis term used alone improved AP_S_ over the detection-only objective but did not match the overall accuracy of the combined frequency and offset terms. Combining all three auxiliary terms gave the best result.

The loss ablation also exposed an interaction among the auxiliary terms. On FLIR and M3FD, the small-target term alone raised AP_S_ to 47.0% and 44.7%, respectively, whereas the frequency and offset terms together yielded higher overall mAP_50_. The full loss improved both mAP_50_ and AP_S_ beyond either partial objective. These results support pairing recall-oriented supervision with alignment regularization in multispectral small-object detection.

Table 10 summarizes the sensitivity to channel pruning and frequency partitioning. The keep ratio in LWPM and the frequency partition in FGAM affected the accuracy–efficiency balance. Aggressive pruning reduced parameters and improved speed but removed small-target contours. A keep ratio close to one preserved accuracy but reduced the efficiency advantage. The three-band spectral descriptor outperformed a low/high-only partition because middle-frequency responses often represent vehicle edges and pedestrian clothing boundaries.

The default keep ratio of 0.65 offered the best compromise between compactness and discriminative detail. Reducing the ratio to 0.45 increased speed to 78 FPS, but FLIR and M3FD mAP_50_ dropped to 90.7% and 88.9%, respectively. Increasing the ratio to 0.80 or removing pruning did not improve accuracy, suggesting that the discarded channels were not merely redundant in computation but also carried noisy or weakly aligned responses. The comparison between three-band and two-band frequency partition further shows that middle-frequency cues should be modeled explicitly; collapsing them into a coarse low/high split weakened the detector even though the parameter count and speed were nearly unchanged.

The auxiliary-loss weights were varied one at a time on the FLIR validation split. Table 11 shows a broad optimum around the selected values rather than a sharp dependence on a single setting. The small-target weight had the clearest effect on AP_S_, whereas an excessive frequency or offset weight reduced overall mAP_50_ by over-constraining intermediate features.

The CAST sampling-grid comparison in Table 12 showed a similar trade-off. A 1×1 grid lacked neighborhood support for local displacement. Expanding the grid from 3×3 to 5×5 produced only a marginal accuracy change while reducing throughput. We therefore used 3×3 for every dataset.

### 5.8. Robustness Under Difficult Conditions

The robustness analysis separates performance by condition rather than averaging all images together. FGCFF-Netachieved the largest margins under night, glare, rain/fog, and tiny-object subsets, where spectral reliability and cross-modal offset prediction are most relevant. Under normal daylight, the margin over efficient visible detectors was smaller. The main benefit of FGCFF-Netis therefore resilience to modality-dependent degradation rather than generic model capacity.

The condition-wise results also explain why average benchmark scores can underestimate the value of multispectral fusion. In normal daytime scenes, visible features are already strong and the incremental benefit of infrared information is moderate. In night, glare, and adverse weather; however, the reliability of each modality changes locally across the image. FGCFF-Netimproved these subsets by using frequency discrepancy as a cue for selecting and aligning modality evidence. The gains on tiny objects and occlusion further show that the detector retained weak local evidence instead of averaging it away during fusion. These results support the use of condition-specific evaluation when assessing lightweight visible–infrared detectors.

## 6. Discussion

The results indicate that frequency-guided fusion is most useful when weak target boundaries must be preserved while modality-specific background noise is suppressed. A simple dual-stream detector increases feature diversity but lacks an explicit mechanism for deciding whether visible texture, infrared thermal contrast, or cross-modal agreement should dominate. FGCFF-Netaddresses this decision progressively. LWPM limits the channel budget before expensive interaction. FGAM applies residual spectral compensation without replacing either modality. CAST converts bidirectional spectral reliability into deformable sampling offsets, allowing small targets to remain spatially coherent. FACA then performs attention-based aggregation conditioned on frequency discrepancy.

The qualitative and activation results support this interpretation. The class activation maps became more object-centered rather than simply stronger. This distinction is important because multispectral detectors can increase confidence by spreading activation over thermal regions that correlate with objects but do not define object boundaries. For small objects, broad activation often causes duplicate boxes or shifted localization. The lower duplicate and localization-error counts in Table 6, together with the improved tiny-object performance in Table 13, indicate that the complete frequency-guided model converts saliency into more precise localization evidence.

The method has several limitations. First, it assumes paired visible and infrared frames with moderate spatial correspondence. CAST tolerates local offsets, but severe temporal desynchronization, parallax, or large viewpoint differences require explicit calibration or temporal alignment. Second, the learned frequency descriptors do not estimate the physical parameters in Equations (1) and (2). Strong visible-band glare may clip texture irreversibly, and heavy fog may attenuate both modalities. Thermal fixed-pattern noise, blooming, low emissivity contrast, and hot backgrounds can also create infrared responses that resemble targets. Third, neither spectral compensation nor deformable sampling can recover a fully occluded object without contextual evidence. Finally, the experiments use public benchmarks and a single matched hardware protocol. Sensor-specific noise, unusual thermal environments, calibration drift, and embedded-device latency require separate validation before safety-critical deployment.

## 7. Conclusions

This study presents FGCFF-Net, a lightweight frequency-guided detector for visible–infrared small objects. The framework prunes redundant channels, compensates spectral discrepancies, adjusts local sampling positions, and aggregates calibrated cross-modal features. Under the matched evaluation protocol, it improved detection accuracy and small-object AP on FLIR, M3FD, LLVIP, and KAIST while running at 68 FPS with 14.8M parameters. This throughput is lower than that of several compact YOLO baselines, so the contribution is an accuracy-oriented trade-off rather than maximum speed. The current evidence supports frequency-guided alignment for paired sensors with moderate spatial correspondence; severe desynchronization, occlusion, and sensor-specific degradation remain open deployment constraints.

## Figures and Tables

**Table 1:** Brief terminology used in the proposed framework.

Term	Meaning in This Study
Cross-modal fusion	Feature-level combination of paired visible and infrared evidence for a common detection output.
Frequency guidance	Conditioning of feature compensation or attention on pooled low-, middle-, and high-frequency responses.
Frequency-aware attention	Channel or spatial attention whose logits include a descriptor of visible–infrared spectral discrepancy.
Deformable alignment	Learned displacement of convolutional sampling locations to compensate for local cross-modal offset.
Small object	A ground-truth box with area below $32^{2}$ pixels after resizing the image to 640×640; the additional “tiny” bin is below $16^{2}$ pixels.

**Table 2:** Dataset protocol and evaluation setting.

Dataset	Primary Scene	Modalities	Pairs	Classes	Train/Val/Test	Main Metric	Detection Emphasis
FLIR	Road driving	RGB/TIR	14,452	3	8862/1366/4224	mAP_50_, AP_S_	Distant vehicles and pedestrians
M3FD	Multi-scenario traffic	RGB/TIR	4200	6	2940/420/840	mAP_50_, mAP_50:95_	Weather, scale, and category diversity
LLVIP	Low-light surveillance	RGB/TIR	15,488	1	12,025/1732/1731	AP_50_, AP_S_	Nighttime pedestrians
KAIST	Urban driving	RGB/TIR	95,328	1	50,172/8340/36,816	log-average MR, mAP_50_	Day/night pedestrian detection

**Table 3:** Baseline taxonomy. The table groups representative detectors by mechanism so that performance differences can be interpreted beyond publication year.

Category	Method	Core Mechanism	Fusion Level	References
Two-stage detector	Faster R-CNN	Region proposals and RoI features	RGB/IR variants	[59]
One-stage detector	SSD	Dense default boxes	RGB/IR variants	[39]
One-stage detector	RetinaNet	Focal loss for dense detection	RGB/IR variants	[35]
One-stage detector	YOLOv3/v4	Real-time anchor-based detection	RGB/IR variants	[40,41]
Efficient detector	YOLOv7/Gold-YOLO	Trainable reparameterization and gather-distribute design	RGB/IR variants	[42,43]
Recent detector	YOLOv9/YOLOv10	Programmable gradients and end-to-end real-time detection	RGB/IR variants	[44,45]
Detection transformer	DETR/Deformable DETR/DINO	Query-based end-to-end detection	RGB/IR variants	[36,37,38]
Real-time transformer	RT-DETR/RT-DETRv2	Efficient hybrid encoder and real-time transformer decoding	RGB/IR variants	[46,47]
Classical multispectral	IAF R-CNN, CIAN, AR-CNN, MBNet	Illumination, interaction, weak alignment, modality balance	Middle/high-level fusion	[1,2,3,4]
Refinement fusion	CFR, GAFF, UCGM	Cyclic refinement, attentive guidance, uncertainty guidance	Multi-level fusion	[18,19,60]
Transformer fusion	CFT, ICAFusion, C^2^Former, CAGTDet	Cross-attention, calibration, cascade alignment	Multi-level fusion	[5,6,7,8]
Recent lightweight/frequency	MOD-YOLO, M^2^FNet, FMCFNet, EI^2^Det, EMOD	Lightweight fusion, frequency mining, edge/illumination guidance	Multi-level fusion	[9,10,11,12,50]
Ours	FGCFF-Net	Lightweight pruning, frequency-guided alignment, deformable sampling, FACA	Progressive fusion	This work

**Table 4:** Main comparison on FLIR and M3FD. Params, FLOPs, and FPS were measured under the same protocol.

Method	Type	Params (M)	FLOPs (G)	FPS	FLIR		M3FD		
					mAP_50_	AP_S_	mAP_50_	mAP_50:95_	AP_S_
Faster R-CNN	Two-stage	41.3	184.5	18	78.6	31.2	76.4	42.7	28.5
RetinaNet	One-stage	36.5	112.7	33	79.1	32.6	77.8	43.9	29.1
SSD	One-stage	24.1	62.4	71	74.8	27.7	73.5	39.8	25.4
YOLOv3	One-stage	61.5	154.7	42	80.2	33.5	78.0	44.1	30.2
YOLOv5s	One-stage	7.2	16.5	96	81.5	35.4	79.3	45.7	31.8
YOLOv7	One-stage	36.9	104.7	59	83.1	37.6	80.7	47.2	33.5
YOLOv8 s	One-stage	11.2	28.6	82	83.6	38.4	81.1	47.8	34.1
YOLOv9 s	One-stage	12.4	31.8	75	84.4	39.1	81.8	48.6	35.0
YOLOv10 s	One-stage	8.0	24.9	88	84.7	39.6	82.4	49.0	35.6
RT-DETR-R18	Transformer	20.0	60.1	64	85.5	40.3	83.0	49.8	36.4
DINO-R50	Transformer	47.0	279.2	16	86.0	41.1	84.1	50.5	37.0
Early-fusion YOLOv8	Fusion	11.5	29.3	76	85.1	39.9	83.7	50.1	36.8
Middle-fusion YOLOv8	Fusion	15.9	43.7	61	86.8	41.7	84.9	51.3	38.6
IAF R-CNN	Multispectral	45.6	190.3	15	84.5	38.8	82.6	48.7	35.9
CIAN	Multispectral	39.8	153.2	22	85.3	40.5	83.4	49.4	37.2
AR-CNN	Multispectral	44.1	167.9	20	85.8	41.0	84.0	49.9	37.9
MBNet	Multispectral	42.9	161.8	23	86.1	41.5	84.2	50.2	38.1
CFT	Transformer fusion	37.5	122.4	45	86.4	41.8	84.7	50.8	38.6
ICAFusion	Transformer fusion	42.1	136.9	38	88.7	42.9	86.5	52.6	39.7
C^2^Former	Transformer fusion	48.7	155.6	31	89.4	43.8	87.0	53.1	40.6
MOD-YOLO	Lightweight fusion	18.6	47.2	57	88.9	43.1	86.9	52.8	40.1
M^2^FNet	Fusion	41.8	129.5	34	89.1	43.5	87.5	53.4	40.8
CAGTDet	Transformer fusion	58.3	178.4	26	89.8	44.2	88.0	54.0	41.5
EI^2^Det	Illumination-guided	24.6	61.8	41	90.2	44.8	88.9	54.7	42.3
EMOD	Efficient fusion	21.9	53.6	52	90.5	45.2	89.2	55.0	42.6
DMPFDet	Deformable fusion	54.2	169.1	24	91.0	45.9	89.8	55.7	43.5
FGCFF-Net	Ours	**14.8**	**38.4**	**68**	**92.4**	**47.9**	**90.7**	**57.1**	**45.4**

*Note:* Bold numerical entries identify FGCFF-Net; they do not necessarily indicate the best value in each column.

**Table 5:** Main comparison on LLVIP and KAIST. Lower MR is better.

Method	Type	LLVIP			KAIST		
		AP_50_	AP_75_	AP_S_	mAP_50_	MR(%) ↓	AP_S_
YOLOv5s	One-stage	92.1	66.4	54.2	69.8	11.86	28.4
YOLOv7	One-stage	93.4	68.7	56.8	71.6	10.74	30.6
YOLOv8 s	One-stage	94.0	69.5	57.9	72.8	10.28	31.5
RT-DETR-R18	Transformer	94.7	70.4	58.6	73.2	9.96	32.1
IAF R-CNN	Multispectral	93.6	68.9	56.7	72.4	10.15	31.8
CIAN	Multispectral	94.2	70.1	58.0	73.5	9.72	33.0
AR-CNN	Multispectral	94.6	70.8	58.8	74.1	9.38	33.4
MBNet	Multispectral	95.0	71.3	59.5	74.8	8.92	34.1
CFT	Transformer fusion	95.5	72.6	60.4	75.5	8.51	35.0
ICAFusion	Transformer fusion	96.4	74.5	61.9	77.0	7.86	36.7
C^2^Former	Transformer fusion	97.1	75.6	62.8	77.8	7.42	37.6
MOD-YOLO	Lightweight fusion	96.6	74.9	62.2	77.2	7.60	37.1
M^2^FNet	Fusion	97.0	75.5	62.7	77.6	7.48	37.5
CAGTDet	Transformer fusion	97.5	76.4	63.4	78.4	6.98	38.6
EI^2^Det	Illumination-guided	97.9	77.1	64.1	78.8	6.54	39.2
EMOD	Efficient fusion	98.0	77.4	64.3	79.1	6.41	39.5
DMPFDet	Deformable fusion	98.2	77.9	64.7	79.6	6.09	40.0
FGCFF-Net	Ours	**98.5**	**78.6**	**65.4**	**80.1**	**5.72**	**41.3**

*Note:* Bold numerical entries identify FGCFF-Net. The downward arrow (↓) indicates that a lower log-average miss rate (MR) is better.

**Table 6:** Quantitative error analysis. Misses are reported per 1000 small-object ground truths; duplicates and localization errors are reported per 1000 predictions; background false positives (FP) are reported per image.

Dataset	Method	Missed Small Objects	Duplicate Detections	Background FP/Image	Localization Errors
FLIR	Middle-fusion baseline	184	78	0.42	146
FLIR	FGCFF-Net	**132**	**55**	**0.29**	**102**
M3FD	Middle-fusion baseline	201	84	0.47	161
M3FD	FGCFF-Net	**149**	**61**	**0.33**	**114**

*Note:* Bold numerical entries identify FGCFF-Netwithin each dataset. Lower values are better for all four error categories.

**Table 7:** Scale-wise AP_50_ analysis. XS: area $<16^{2}$ pixels; S: $16^{2}$–$32^{2}$; M: $32^{2}$–$96^{2}$; L: ≥$96^{2}$, after resizing to 640×640.

Method	Statistic	FLIR AP_50_				M3FD AP_50_			
		XS	S	M	L	XS	S	M	L
Middle-fusion baseline	AP	23.8	41.7	63.5	78.9	26.9	38.6	66.1	81.2
FGCFF-Net	AP	**33.4**	**47.9**	**67.2**	**80.5**	**36.8**	**45.4**	**69.6**	**82.7**
Gain	Percentage points	+9.6	+6.2	+3.7	+1.6	+9.9	+6.8	+3.5	+1.5

*Note:* Bold numerical entries identify FGCFF-Net.

**Table 8:** Module ablation on four datasets.

Configuration	Params (M)	FLOPs (G)	FLIR	M3FD	LLVIP	KAIST	AP_S_ Avg.	MR ↓
			mAP_50_	mAP_50_	AP_50_	mAP_50_	FLIR/M3FD/KAIST	KAIST
Baseline middle fusion	15.9	43.7	86.8	84.9	95.2	73.6	37.4	9.91
+LWPM	13.7	36.9	87.5	85.7	95.8	74.4	38.2	9.35
+LWPM +FGAM	14.1	37.6	88.6	86.8	96.6	75.6	39.6	8.53
+LWPM +FGAM +FACA	14.6	38.1	90.1	88.2	97.4	77.2	41.8	7.31
+LWPM +FGAM +FACA +CAST	14.8	38.4	91.2	89.4	98.0	78.6	43.5	6.28
Full objective	**14.8**	**38.4**	**92.4**	**90.7**	**98.5**	**80.1**	**44.9**	**5.72**

*Note:* Bold numerical entries identify the full model trained with the complete objective. The downward arrow (↓) indicates that a lower KAIST log-average miss rate (MR) is better.

**Table 9:** Loss ablation on FLIR and M3FD.

Objective	FLIR mAP_50_	FLIR AP_S_	M3FD mAP_50_	M3FD AP_S_
$L_{\det}$ only	91.2	45.8	89.4	43.5
+$L_{\mathrm{freq}}$	91.7	46.6	89.9	44.2
+$L_{\mathrm{freq}}+L_{\mathrm{off}}$	92.0	47.1	90.3	44.8
+$L_{\mathrm{small}}$ only	91.8	47.0	90.0	44.7
Full loss	**92.4**	**47.9**	**90.7**	**45.4**

*Note:* Bold numerical entries identify the full-loss configuration.

**Table 10:** Sensitivity to LWPM keep ratio and frequency partition.

Setting	Keep Ratio	Bands	Params (M)	FPS	FLIR mAP_50_	M3FD mAP_50_
Aggressive pruning	0.45	Low/Mid/High	11.9	78	90.7	88.9
Moderate pruning	0.55	Low/Mid/High	13.2	72	91.8	90.0
Default	**0.65**	Low/Mid/High	**14.8**	**68**	**92.4**	**90.7**
Weak pruning	0.80	Low/Mid/High	17.6	59	92.3	90.6
No pruning	1.00	Low/Mid/High	21.4	47	92.1	90.3
Default without mid band	0.65	Low/High	14.6	69	91.6	89.8

*Note:* Bold numerical entries identify the default configuration, rather than the minimum parameter count or maximum FPS.

**Table 11:** One-factor sensitivity of the auxiliary-loss weights on FLIR. The default value in each group is bold.

Varied Weight	Value	mAP_50_	AP_S_
$\lambda_{f}$	0.10	92.0	47.3
	**0.20**	**92.4**	**47.9**
	0.40	92.1	47.6
$\lambda_{o}$	0.025	92.1	47.4
	**0.050**	**92.4**	**47.9**
	0.100	92.2	47.7
$\lambda_{s}$	0.20	92.0	47.1
	**0.40**	**92.4**	**47.9**
	0.60	92.1	47.8
$\lambda_{r}$	$5\times 10^{-5}$	92.2	47.6
	$1\times 10^{-4}$	**92.4**	**47.9**
	$2\times 10^{-4}$	92.0	47.3

**Table 12:** Sensitivity to the deformable sampling grid.

Sampling Grid	FPS	FLIR mAP_50_	FLIR AP_S_	M3FD mAP_50_	M3FD AP_S_
1×1	72	91.4	46.7	89.7	44.2
3×3	**68**	**92.4**	**47.9**	**90.7**	**45.4**
5×5	54	92.5	48.0	90.8	45.5

*Note:* Bold numerical entries identify the selected 3×3 sampling grid, rather than the optimum of every metric.

**Table 13:** Condition-wise AP_50_ (%) on the FLIR and M3FD evaluation subsets.

Method	Day	Night	Glare	Rain/Fog	Tiny Objects	Occlusion
YOLOv8 s	86.5	78.4	76.9	75.8	38.4	70.5
ICAFusion	90.2	84.7	83.6	82.5	42.9	75.8
C^2^Former	90.7	85.5	84.2	83.1	43.8	76.4
EMOD	91.1	86.3	85.0	84.5	45.2	77.6
DMPFDet	91.4	87.0	85.8	85.2	45.9	78.2
FGCFF-Net	**92.6**	**89.4**	**88.1**	**87.6**	**47.9**	**80.5**

*Note:* Bold numerical entries identify FGCFF-Net.

## Data Availability

The datasets used in this study are publicly available from the following sources: FLIR ADAS, https://www.flir.com/oem/adas/dataset/ (accessed on 23 September 2026); M3FD, https://github.com/YulongTan/TarDAL (accessed on 23 September 2026); LLVIP, https://bupt-ai-cz.github.io/LLVIP/ (accessed on 23 September 2026); and KAIST, https://github.com/SoonminHwang/rgbt-ped-detection (accessed on 23 September 2026). No new dataset was created for this study.

## Abbreviations

The following abbreviations are used in this manuscript:

AP	Average Precision
CAM	Class Activation Map
CAST	Cross-modal Alignment and Small-Target framework
CFFM	Cross-Modal Feature Fusion Module
FACA	Frequency-Aware Cross-modal Aggregator
FGAM	Frequency-Guided Alignment Module
FPS	Frames Per Second
LWPM	Lightweight Channel Pruning Module
mAP	mean Average Precision
MR	Miss Rate
RGB	Red–Green–Blue visible modality
TIR	Thermal Infrared modality
